# Novel Improved Salp Swarm Algorithm: An Application for Feature Selection

**DOI:** 10.3390/s22051711

**Published:** 2022-02-22

**Authors:** Miodrag Zivkovic, Catalin Stoean, Amit Chhabra, Nebojsa Budimirovic, Aleksandar Petrovic, Nebojsa Bacanin

**Affiliations:** 1 Faculty of Informatics and Computing, Singidunum University, Danijelova 32, 11010 Belgrade, Serbia; mzivkovic@singidunum.ac.rs (M.Z.); nebojsa.budimirovic.20@singimail.rs (N.B.); aleksandar.petrovic@singidunum.ac.rs (A.P.); 2 Human Language Technology Research Center, University of Bucharest, 010014 Bucharest, Romania; catalin.stoean@fmi.unibuc.ro; 3 Department of Computer Engineering and Technology, Guru Nanak Dev University, Amritsar 143005, India; amit.cse@gndu.ac.in

**Keywords:** feature selection, swarm intelligence, hybridization, learnheuristics, salp swarm algorithm

## Abstract

We live in a period when smart devices gather a large amount of data from a variety of sensors and it is often the case that decisions are taken based on them in a more or less autonomous manner. Still, many of the inputs do not prove to be essential in the decision-making process; hence, it is of utmost importance to find the means of eliminating the noise and concentrating on the most influential attributes. In this sense, we put forward a method based on the swarm intelligence paradigm for extracting the most important features from several datasets. The thematic of this paper is a novel implementation of an algorithm from the swarm intelligence branch of the machine learning domain for improving feature selection. The combination of machine learning with the metaheuristic approaches has recently created a new branch of artificial intelligence called learnheuristics. This approach benefits both from the capability of feature selection to find the solutions that most impact on accuracy and performance, as well as the well known characteristic of swarm intelligence algorithms to efficiently comb through a large search space of solutions. The latter is used as a wrapper method in feature selection and the improvements are significant. In this paper, a modified version of the salp swarm algorithm for feature selection is proposed. This solution is verified by 21 datasets with the classification model of K-nearest neighborhoods. Furthermore, the performance of the algorithm is compared to the best algorithms with the same test setup resulting in better number of features and classification accuracy for the proposed solution. Therefore, the proposed method tackles feature selection and demonstrates its success with many benchmark datasets.

## 1. Introduction

The fields of big data, cryptography, and computer science in general are all influenced by the domain of optimization and to some extent even somewhat rely on it. The field of optimization is broad and employs a large variety of techniques. Although there is a large number of optimization solutions, in most of the cases there is room for further improvements and new algorithms can lead to better results. What is more, some optimization methods prove to be suitable for a certain class of problems, while others perform better for other types. Consequently, when proposing a new optimization technique, it needs to be thoroughly tested in order to identify its strengths and weaknesses with respect to the solutions’ quality when dealing with different types of problems.

Nature-inspired algorithms have been widely applied in recent years for solving various range mathematical and engineering optimization non-deterministic polynomial hard (NP-hard) problems [1] due to its high robustness and efficiency in exploiting and exploring vast search space domain. Of all nature-inspired approaches, evolutionary algorithms (EA) and swarm intelligence metaheuristics stand out the most and they have been effectively applied to different NP-hard real-world challenges [2,3,4]. The EA approaches conduct a search process by adopting reproduction, crossover and mutation operators from natural evolution, while swarm intelligence mimics collective intelligent behavior of group of organisms from nature such as flock of birds, school of fish, colonies of ants and bees, and so forth. Both families of methods belong to the group of artificial intelligence optimization techniques. Various metaheuristics were reviewed and considered to be improved upon. The most recent from the reviewed set are the grey wolf optimizer (GWO), red deer algorithm (RDA) [5], ant lion optimizer (ALO) [6], grasshopper optimization algorithm (GOA) [7], multi-verse optimizer (MVO) [8], moth-flame optimization algorithm (MFO) [9], social engineering optimizer (SEO) [10], dragonfly algorithm (DA) [11], whale optimization algorithm (WOA) [12], harris hawks optimization (HHO) [13], sine cosine algorithm (SCA) [14]. While the mentioned algorithms have all shown notable improvement performance-wise, none are without shortcomings. In the field of swarm metaheuristics, the primary solutions tend to favor either exploration or exploitation phases. There have been attempts in the domain to initially create a solution that performs equally well in both phases like the elaborate SCA. Nevertheless, even the SCA has undergone modifications and achieved better performance than its original version [15]. Hence, the true potential of the swarm metaheuristics is achieved through hybridization. This modification method relies on the principle of fusing the original algorithm with another. This is usually achieved by incorporating a principle from an algorithm that has better performance for the phase that unfavored by the solution that is improved upon. The dynamic of the field dictates constant improvement and search for new solutions and new ways to improve the existing ones. The reason for the authors to opt to improve SSA is with its robustness while maintaining simplicity. The algorithm is easy to implement and the fine-tuning modifications are even suggested by its author.

The expansion of data availability and computer processing power in recent decades has led to interaction between the fields of nature-inspired metaheuristics and machine learning, which is an artificial intelligence subdomain and as a crucial tool for data science. Machine learning models can be efficiently utilized to find patterns and make predictions from what may appear at first glance uncorrelated huge amounts of data. However, employed large datasets are usually packed with inessential and redundant data negatively influencing machine learning performance regarding computational complexity and accuracy. An attribute of “high-dimensional” is usually associated with such datasets and this phenomena is known as the curse of dimensionallity [16].

Therefore, finding relevant information (features) from large datasets is crucial for tackling the above mentioned issue and it is known as the dimensionality reduction challenge in the modern computer science literature [17]. The process of dimensionality reduction is usually employed in the data pre-processing phase of machine learning and it encompasses two approaches: feature extraction and feature selection. By using feature extraction, new variables are derived from the primary dataset [18], while feature selection chooses a subset of significant variables for further use [19].

The aim of feature selection is to find the most informative subset from high-dimensional datasets by removing redundant and irrelevant features, therefore improving classification and prediction accuracy of machine learning model. According to G. Chandrashekar et al. [20], all feature selection methods can be split into three groups: filter, wrapper and embedded. Wrapper methods utilize learning algorithms to evaluate feature subset by training a model and they are the most efficient, however the most computational demanding as well. Filter methods do not rely on a training system, but apply a measure to assign a score to feature subsets. This group is generally less computationally expensive than the wrapper family, but generates a universal set (not tuned to a particular predictive model) since it does not include model training. Finally, the embedded methods use feature selection as a part of the model construction procedure, that is, algorithms execute feature selection during the model training. The embedded methods are as fast as the filter ones, but more precise. Regarding the computational difficulty, embedded methods are in the middle of wrappers and filters.

Nature-inspired algorithms, especially swarm intelligence metaheuristics [21,22], have been successfully applied as wrapper methods for feature selection in machine learning and this is one point where machine learning and optimization metaheuristics intersect. If there are nf features in a dataset, the total number of $2^{nf}$ subsets exist and, since for high-dimensional datasets nf is typically a large number, this challenge is considered NP-hard. Consequently, regarding the fact that swarm intelligence proved to be a robust and efficient optimizer for solving NP-hard challenges, its application as a wrapper feature selection method is straightforward.

Notwithstanding that many swarm intelligence applications for feature selection can be found by surveying recent literature sources, considering no free lunch (NFL) theorem [23], there is still space for improvements in this domain. The NFL, which proved to be accurate, states that no universal algorithm exists that can solve all optimization problems. Accordingly, an approach that efficiently solves feature selection issues for all datasets does not exist. The NFL theorem motivates researchers to improve and adjust current algorithms or propose new ones, to solve various problems, including feature selection challenge.

Therefore, the motivation behind the proposed study is to try to further enhance feature selection in machine learning by employing an improved salp swarm algorithm (SSA), which was also developed and evaluated for the purpose of this research. The SSA belongs to the family of swarm intelligence metaheuristics and it was proposed in 2017 by Mirjalili et al. [24]. The basic SSA is enhanced by including an additional mechanism and by hybridization with another well-known swarm intelligence metaheuristics.

Guided by established practice from the modern literature, before its application to feature selection, the proposed enhanced SSA is firstly tested and evaluated on a recognized test-bed with challenging instances of functions having 30 dimensions from the Congress on Evolutionary Computation 2013 (CEC2013) benchmark suite [25]. This also allows a direct comparison of the obtained results with the outputs of a large variety of state-of-the-art (SOTA) metaheuristics. Afterwards, it is adapted as a wrapper-based approach for feature selection and validated against 21 well-known datasets retrieved from University of California, Irvine (UCI) repository [26].

The scientific contributions of proposed study can be summed as follows:

- proposed improved SSA algorithm overcomes some observed deficiencies and establishes better performance than original SSA;
- proposed method proves to be promising and competitive with other SOTA metaheuristics according to CEC2013 testing results; and
- compared to other SOTA approaches, improvements in addressing feature selection issue in machine learning in terms of classification accuracy and number of selected features is established.

Based on that stated above, the method proposed in this study tackles the feature selection challenge and demonstrates its success with many benchmark datasets.

The organization of the manuscript is as follows. Section 2 covers some of the most notable SOTA approaches from the domain of swarm intelligence, as well as from the area of hybrid methods between swarm algorithms and machine learning. In Section 3, the original SSA is presented first, then its drawbacks are indicated and finally details of the proposed algorithm are provided. Section 4 and Section 5 present simulations with standard CEC2013 instances along with feature selection experiments including comparative analysis and discussion with other recent SOTA algorithms. Finally, a summary and future research plans are examined in Section 6.

## 2. Related Works

There are several recent good survey studies that present the challenges that appear within feature selection in various fields of machine learning, as well as indicate the most prolific methods to achieve the task. Some very inspiring reads are [20,27], as well as the more recent work [28]. These also thoroughly present the complexity of the feature selection task, the manner in which the dimensionality reduction can be achieved for various datasets, ideas that are also marginally discussed in the introduction section of the current article. Another work that also presents a survey for the same problem is [29]. This study especially concentrates on evolutionary computation approaches for achieving the goal, so it is better linked with the current work. A review of studies for feature selection that is further narrowed only on methodologies involving swarm intelligence algorithms is found in [30].

The two most popular evolutionary computation approaches in feature selection are genetic algorithms (GAs) and particle swarm optimization (PSO), and for both there is an increasing trend in the number of studies using them in the last couple of decades [29]. They are both applied in wrapper approaches beside various classification algorithms, like support vector machines [31,32,33], K-nearest neighbor [34,35,36], artificial neural networks [37,38], decision tree [39] and so forth.

In [31], a regression real-world task regarding combustion processes in industry is considered, where support vector regression is actually employed for getting an optimal carbon monoxide concentration in the exhaust gases based on other characteristics. Besides a GA for feature selection, two more methods from Bayesian statistics are tested, but the GA approach proves to be superior. Another case of successful combination between a GA and SVM for classification is presented in [32], where the GA is used both for feature selection and for fine tuning the parameters of the SVM. In [33], dataset with medical microscopical images is considered and features are first extracted from these and they are further reduced by feature selections and eventually an SVM is applied for achieving automated diagnosis.

In [34], a bees inspired optimization algorithm is used as the metaheuristics that takes care of optimization, several benchmark datasets are used and the results are compared to cases when a GA, a PSO or an ant colony optimization are used. The approach in [36] integrates an evolutionary algorithm with a local search technique and the authors claim very good performance for medium- to large-sized datasets.

In [37], a real-world credit dataset is collected at a Croatian bank and the GA combined with ANN is applied to it and then further tested on a UCI database. Applications to medical data are presented in [38], where various classifiers (SVM, artificial neural networks, K-nearest neighbor, linear regression) are optimized via a genetic algorithm as concerns both parameter optimization and feature selection.

Finally, in [39] an application to medical images performs, as in [33] above, feature extraction and then feature selection is performed using a GA. Various classifiers like SVM, ANN and decision tree are used for the final prediction. Another example of feature selection tackled by swarm intelligence is [40], where the PSO algorithm is validated and improved upon with a innovative mechanism of initialization and the update process of solutions with the 20 popular datasets.

SSA has also been used to address the feature selection problem. Some of the efficiently improved cases of the basic SSA include the solution of feature weighting with the minimum distance problem [41], the problem of feature selection solving through hybridization with the opposition based learning heuristics [42], and the improvement of accuracy, reliability and the convergence time for the problem of feature selection with the introduction of the inertia weight control parameter [43]. SSA has also been successfully modified and applied in other application domains recently, such as green home health care routing problem [44], health care supply chain [45], crop disease detection [46] and power systems unit commitment task [47], to name the few.

Nature is the source of inspiration in the case of swarm intelligent algorithms. The benefit for the machine learning techniques derives from the good compatibility with the main principle of swarm intelligence of employing an immense amount of units individually incapable of solving the problem. This sort of algorithms are often applied by themselves for the reason of their well known exceeding performance. Furthermore, their full potential is reached by incorporating hybridization techniques. The real world application of swarm intelligence solutions is vast from the clustering, node localization, and preserving of energy in wireless sensor networks [48,49,50,51], through to the scheduling problem with cloud tasks [2,52], the prediction of COVID-19 cases based on machine learning [53,54], MRI classification optimization [55,56], text document clustering [57], and the optimization of the artificial neural networks [58,59,60,61].

## 3. Proposed Method

This section first introduces basic details of the original SSA metaheuristics. Afterwards, the observed drawbacks of the basic version are elaborated and mechanisms that are able to overcome its deficiencies are proposed. Finally, solutions for improving SSA are put forward.

### 3.1. Basic Salp Swarm Algorithm

The SSA [24] algorithm was motivated by the group of animals called salp, which are aquatic, small, barrel-shaped and transparent. The individual units of this specimen bind together with the goal of finding the safest paths in finding food sources. These interesting creatures link up one behind another forming a chain.

The first unit in the chain is the leader and its behavior models exploration and exploitation of the optimization algorithm search process. The leader decides where the group will go in search for paths and food in its area. The leader’s position is changed towards the direction of the food source, that represents the current best solution.

The units’ positions in *D*-dimensional search space are mathematically described as a two-dimensional matrix labeled *X*, while the food source (current best solution) is labeled as *F*. The following function updates the leader’s position in the *j*-th dimension [24]:

(1) $$x_{j}^{1}=\left\{\begin{array}{c} F_{j}+c_{1}\left(\left(ub_{j}-lb_{j}\right)c_{2}+lb_{j}\right),\;c_{3}\geq 0.5\\ F_{j}-c_{1}\left(\left(ub_{j}-lb_{j}\right)c_{2}+lb_{j}\right),\;c_{3}<0.5, \end{array}\right.$$

the $x^{1}$ denotes leader, $F_{j}$ represents the position of the current best solution (food source), the upper and lower search space boundaries in the *j*-th dimension are, respectively, $ub_{j}$ and $lb_{j}$, while $c_{1}$, $c_{2}$ and $c_{3}$ denote pseudo-random numbers drawn from the interval [0,1].

The parameters $c_{2}$ and $c_{3}$ determine the step size and dictate whether the position of the new solution will be generated towards negative or positive infinity. However, the most important parameter is considered to be $c_{1}$ due to the reason that it directly influences the exploration and exploitation balance, which is one of the most important factors that influence search process efficiency. The $c_{1}$ is calculated as [24]:

(2) $$c_{1}=2e^{-{\left(\frac{4l}{L}\right)}^{2}},$$

where the current iteration is represented as *l* and the maximum iterations in a run are denoted as *L*.

The position of followers is updated with the following equation that represents Newton’s law of motion [24]:

(3) $$x_{j}^{i}=\frac{1}{2}at^{2}+V_{0}t,$$

where $x_{j}^{i}$ denote *i*-th follower in the *j*-th dimension and i≥2. Annotation *t* represents time and $a=\frac{V_{final}}{V_{0}}$, where $V=\frac{x-x_{0}}{t}$, and the initial speed is $V_{0}$.

Due to the fact that time in any optimization process is modeled as iteration, the disparity between iterations is 1 and $V_{0}=0$ at the beginning, Equation (3) can be reformulated as:

(4) $$x_{j}^{i}=\frac{1}{2}\left(x_{j}^{i}+x_{j}^{i-1}\right)$$

### 3.2. Cons of the Original Algorithm and Proposed Improved Approach

It is a common case for the basic optimization algorithms to have certain deficiencies and that is also the case with the SSA. Noticed cons of the basic SSA can be summarized as follows: insufficient exploration, average exploitation power (conditional drawback) and intensification-diversification trade-off.

In general, any optimization algorithm can be improved by applying small modifications, for example, minor changes made to the search equation, additional mechanisms, and/or significant changes by hybridization with other algorithm. For the purpose of this study, basic SSA was improved by including novel mechanism, as well as hybridization with another well-known optimization metaheuristics.

Based on the findings from previous research [62,63], as well as on extensive simulations with challenging CEC2013 benchmark instances [25] that were conducted for the purpose of this study, it was discovered that the diversification process of basic SSA exhibits some deficiencies, which leads to the inappropriate intensification-diversification balance, that is on average dis-balanced towards exploitation.

First of all, the SSA exploration is controlled only by dynamic parameter $c_{1}$ according to Equation (2) and at the beginning of a run it is shifted towards exploration, while at later iterations it slides towards exploitation. However, this mechanism is applied only to the leader *F* (current best solution) and the whole search process to some extent depends on the luck. Followers are updated according to Equation (4), which is essentially exploitation between its previous and current positions. If the algorithm was lucky and manages to find a region of the search space where the optimum solution resides, then the search process will eventually converge and satisfying solutions’ quality will be obtained. Conversely, the search will stuck in sub-optimal regions and best solutions will be located far from global optimum at the end of a run.

Therefore, a solution for the above mentioned issue would be to improve exploration in early iterations. For achieving this goal, an exploration replacement mechanism is incorporated into the basic SSA in the following way: in the first rmp iterations, the wrs worst solutions from population are rejected and renewed with randomly generated solutions within upper and lower bounds of the search space according to expression:

(5) $$x_{j}=lb_{j}+\left(ub_{j}-lb_{j}\right)\cdot rnd,$$

where rnd is pseudo-random number drawn from a uniform distribution.

The same expression is utilized in the initialization phase, where a starting random population is generated. This mechanism introduces two additional control parameters: replacement mechanism point (rmp), that determines when (in terms of *l*) the replacement mechanism will be triggered and worst replaced solutions (wrs) that controls the number of worst solutions that will be replaced with random ones. If rmp=L, then the enhanced exploration will be performed throughout the whole run, similarly if rmp=0, then the SSA search will executed as in its basic version.

By further analysis of the original SSA, it was also determined that the exploitation procedure with the followers (Equation (4)) is relatively simple depending on their current and previous positions. To overcome this, hybridization with another recently proposed metaheuristics, the SCA [14] is performed. In each iteration, the followers are updated either by using basic SSA equation (Equation (4), or SCA search expression for and individual *i* and component *j*:

(6) $$x_{j}^{i}=\left\{\begin{array}{c} x_{j}^{i}+r_{1}\cdot sin\left(r_{2}\right)\cdot \left|r_{3}P_{j}-x_{j}^{i}\right|,\;r_{4}<0.5\\ x_{j}^{i}+r_{1}\cdot cos\left(r_{2}\right)\cdot \left|r_{3}P_{j}-x_{j}^{i}\right|,\;r_{4}\geq 0.5, \end{array}\right.$$

where $r_{1}$, $r_{2}$, $r_{3}$ and $r_{4}$ are four randomly generated values from the interval [0,1], $P_{j}$ represents the *j*-th component of random individual from population, || indicates the absolute value and sin and cos are standard trigonometric functions.

Similarly, as the original SSA, the SCA employs the following formula to adjust intensification-diversification balance:

(7) $$r_{1}=a-l\frac{a}{L},$$

where the parameter *a* represents a constant.

To control whether the followers’ position will be updated using basic SSA or SCA search, pseudo-random number ϕ is used, as it is shown in Algorithm 1.

**Figure 1:**
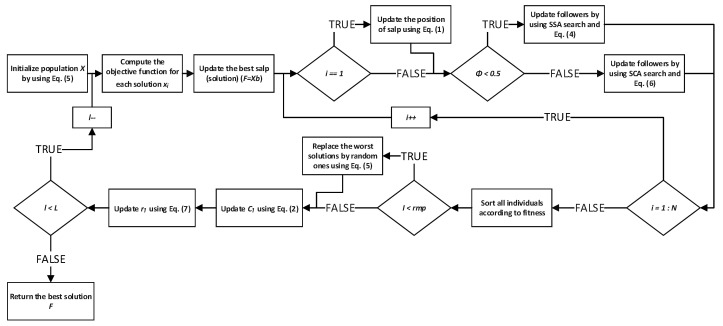
Flowchart of the proposed SSARM-SCA algorithm.

Encouraged with the introduced modifications, proposed enhanced SSA is named SSA with replacement mechanism and SCA search-SSARM-SCA. Its pseudo-code is shown in Algorithm 1. The flowchart of the algorithm is shown in Figure 1.
**Algorithm 1:** Pseudocode of SSARM-SCA.Initialize population *X* by using Equation (5)**repeat**  Compute the objective function for each solution $x_{i}$  Update the best salp (solution) (F=Xb)  **for** i=1:N **do**   **if** i==1 **then**     Update the position of salp using Equation (1)   **end if**   **if** ϕ<0.5 **then**     Update followers by using SSA search and Equation (4)   **else**     Update followers by using SCA search and Equation (6)   **end if**  **end for**  Sort all individuals according to fitness  **if** l<rmp **then**   Replace wrs worst solutions by random ones using Equation (5).  **end if**  Update $c_{1}$ using Equation (2)  Update $r_{1}$ using Equation (7)**until**(l<L)Return the best solution *F*.

### 3.3. Complexity and Limitations of Proposed Method

The most computationally expensive operation during metaheuristics algorithm’s execution is fitness function valuation (FFE). Accordingly, as established in the most relevant and contemporary computer science publications, the complexity of the algorithm is measured in terms of utilized FFEs [64].

Complexity of both basic SSA and the proposed SSARM-SCA algorithms is the same: O(NP)+O(2·NP·T), where NP denotes the number of solutions in the population, while *T* represents the number of iterations. The proposed algorithm in each iteration performs the search either by utilizing the SSA or SCA search equations. In the first rmp iterations the wrs solutions are replaced by pseudo-random solutions, however this does not add additional costs in terms of FFE, as all solutions in the population are being evaluated at the beginning of each iteration.

When the FFE is being considered, the proposed SSARM-SCA algorithm is not more complex than the basic SSA metaheuristics. The algorithm is slightly more complex if the number of floating point operations is taken into account, however this can be disregarded in comparison to FFE, and therefore it is not relevant for the algorithm’s complexity.

## 4. Validation of the Proposed Method for Standard CEC2013 Benchmarks

Following good practice from modern literature, the proposed SSARM-SCA is first tested on challenging CEC2013 benchmark instances [25] with 30 dimensions (D=30) before being adapted for the practical feature selection challenge. With the goal of making comparative analysis with other SOTA approaches, which results are published in the recent papers, the same experimental conditions in terms of control parameters as in [65] are kept.

The CEC2013 benchmark suite contains 28 functions that are split into three groups based on its characteristics. Test instances from 1 to 5 are unimodal, benchmarks from 6 to 20 are multimodal, and finally, test bed from 21 to 28 belongs to the category of composite functions. Functions’ details employed in simulations are given in Table 1.

Besides the proposed method and original SSA, for the purpose of comparative analysis, all methods shown in [65] are also implemented and evaluated. All algorithms are tested with 50 individuals in population N=50 and the number of fitness function evaluations maxFFEs of $3\times 10^{5}$ is set as termination condition as in [65].

The SSARM-SCA is compared to practical genetic algorithm (RGA) [66], gravitational search algorithm (GSA) [67], disruption GSA (D-GSA) [68], black hole GSA (BH-GSA) [69], clustered GSA (C-GSA) [70] and attractive repulsive GSA (AR-GSA) [65].

Specific SSARM-SCA control parameters are set as follows: $rms=3\times 10^{2}$ according to expression maxFFEs/1000 and wrs=10 by using formula N/5. Values for these parameters are determined empirically. Dynamic parameter $c_{1}$ for original SSA and SSARM-SCA are adjusted according to Equation (2) and parameter $r_{1}$ of SSARM-SCA is adjusted throughout the run by expression (7). It is noted that in those expressions instead of *l* and *L*, the FFEs and maxFFEs are used, respectively. Other methods implemented for the purpose of comparison are tested with the control parameters suggested in [65].

All algorithms are executed in 51 independent runs and the following metrics in terms of objective function values are captured: best, median, worst, mean and standard deviation. Comparative analysis results are split into three tables based on the function types as follows: Table 2 show results for unimodal, Table 3 presents metrics for multimodal and Table 4 depicts results for composite CEC2013 instances. The best results for each metrics are marked bold in all tables.

First of all, obtained results for all methods for the purpose of this study are similar as in [67], therefore this research validates results reported in [67]. From the comparative analysis results superiority of proposed SSARM-SCA can be unambiguously determined. For most of the benchmarks, including all three types (unimodal, multimodal and composite) in average, the SSARM-SCA obtains the best results for all four indicators among all other SOTA metaheurisitcs. Specifically, when comparing to the original SSA, improvements in terms of convergence speed and results’ quality are substantial.

**Figure 2:**
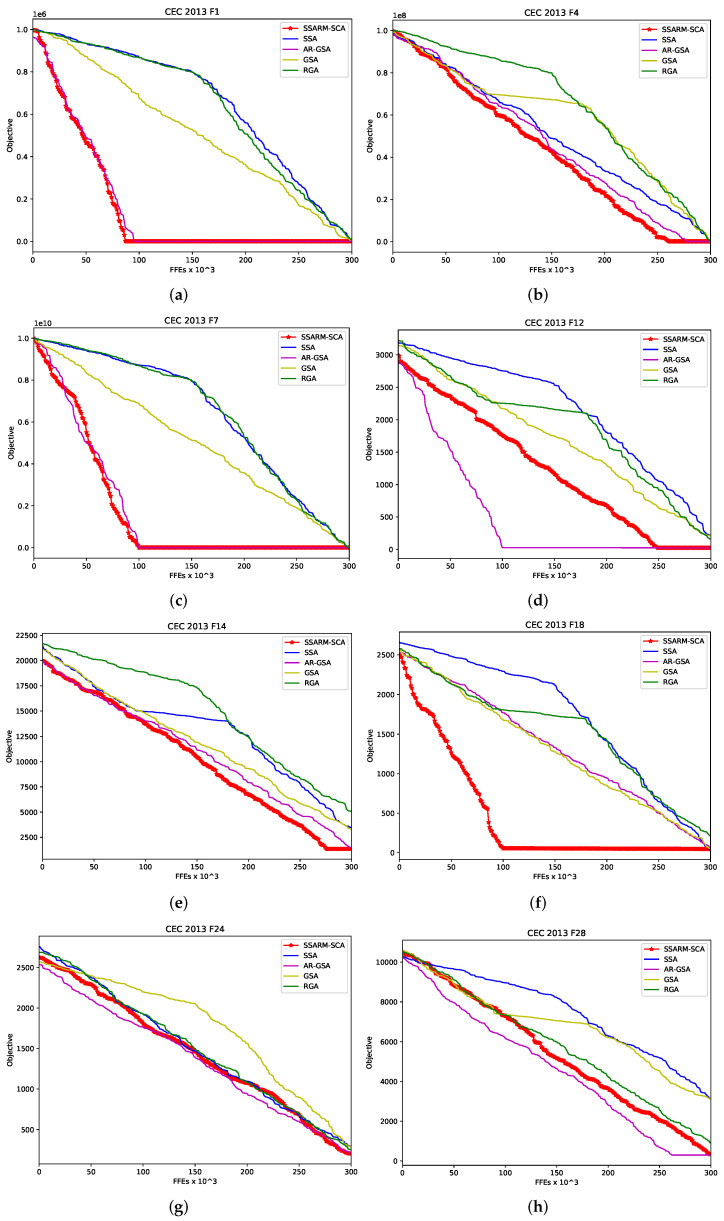
Convergence speed comparison for 8 CEC2013 instances-proposed SSARM-SCA vs. other approaches. (**a**) SSARM-SCA vs. others-CEC2013 F1. (**b**) SSARM-SCA vs. others-CEC2013 F4. (**c**) SSARM-SCA vs. others-CEC2013 F7. (**d**) SSARM-SCA vs. others-CEC2013 F12. (**e**) SSARM-SCA vs. others-CEC2013 F14. (**f**) SSARM-SCA vs. others-CEC2013 F18. (**g**) SSARM-SCA vs. others-CEC2013 F24. (**h**) SSARM-SCA vs. others-CEC2013 F28.

More insights regarding the convergence speed can be obtain from Figure 2. In the presented figure, convergence speed graphs for some methods included in analysis for 2 unimodal (F1 and F4), 4 multimodal (F7,F12,F14 and F18) and 2 composite (F24 and F28) benchmarks are generated. Provided graphs validate clear improvements of proposed SSARM-SCA over original SSA and other SOTA methods in terms of convergence.

However, to more objectively determine the robustness and efficiency of one approach over others, results should also be compared in terms of statistical tests. For that reason, the Friedman test [71,72], as the primary method for doing as alongside the ranked two-way analysis of variances of the proposed method and other implemented methods for the research, was conducted.

The results achieved by the 8 implemented algorithms over the 28 functions from the CEC2013 benchmark set, including the Friedman and the aligned Friedman test, are presented in the Table 5 and Table 6, respectively.

As observed in Table 6, the proposed SSARM-SCA outperformed all of the other candidates, as well as the basic SSA which averaged the ranking of 133.463. Proposed SSARM-SCA obtained an average ranking of 56.838.

Furthermore, the research [73] provides grounds for the possible improvement in terms of performance in comparison with the $\chi^{2}$ value. Hence, the Iman and Davenport’s test [74] is used as well. The results of this test are summarized in Table 7.

The results show a value of $2.230\times 10^{1}$, which demonstrates significantly better results than the *F*-distribution critical value ($F\left(9,9\times 10\right)=2.058\times 10^{0}$). Additionally, the null hypothesis is rejected by Iman and Davenport’s test. The Friedman statistics fared with the score of ($\chi_{r}^{2}=1.407\times 10^{1}$) resulting in better performance than the *F*-distribution critical value at the level of significance being α=0.05.

The final conclusion is that the null hypothesis can be rejected and that the proposed SSARM-SCA is clearly the best of its competitors.

The rejection of the null hypothesis by both statistical tests performed is followed by the next type of test, Holm’s step-down method which is a non-parametric post-hoc method. The findings of such experiments are displayed in Table 8.

The *p* value is the main sorting reference for all the methods and they are compared against the α/(k−i). The *k* denote the degree of freedom while the *i* shows the number of the algorithm, respectively.

This research utilized α parameter at the levels of 0.05 and 0.1. It should be noted that the values of *p* parameter are displayed in scientific notation.

The summary of testing with Holm’s method by the results provided in the Table 8 stands to prove that the improvement has been achieved for the subjected solution in case of both levels of significance.

## 5. Feature Selection Experiments

The feature selection belongs to the group of binary problems, hence the well-known V-shaped transfer function was used for mapping continuous search space variables to discrete values 0 and 1. Therefore, if a dataset consists of nf feature, one solution is represented as a binary array of length nf. This is how the proposed SSARM-SCA was adapted for this problem and for the sake of distinguishing binary version from its respecting continuous version it is referenced as the bSSARM-SCA.

Efficiency of proposed method for feature selection challenge was compared to SOTA metaheuristics presented in [22]. For that reason similar experimental conditions as in [22] were established. However, instead of using L=70 with N=8 as in [22], the maxFFEs was used as termination condition and it was set to 560 (N·L). This approach is more reasonable since different optimization algorithms consume different number of FFEs in each iteration and respecting the fact that the FFE is the most expensive calculation in optimization process. The other SSARM-SCA control parameters were as follows: rms=56 according to formula maxFFEs/10 and wrs=2 by using expression round(N/3).

The bSSARM-SCA performance was tested on the 21 UCI datasets which are often used for bench-marking (Table 9). All datasets are split into training and testing using train_test_split rule in proportion 80%:20%. Each solution’s fitness is calculated on the training set by utilizing nearest neighbors (KNN) classifier and the following fitness function *F* as in [22]:

(8) $$F=\alpha E_{R}\left(D\right)+\beta \frac{\left|R\right|}{\left|C\right|},$$

where the $E_{R}\left(D\right)$ represents the error-rate of classification, the selected features number represented as *R*, and lastly the *C* shows the sum of all features. The α and β are parameters that establish relative influence of the $E_{R}\left(D\right)$ and *R* to the fitness function and they sum to 1 (α=1−β).

From the formulated fitness function it can be seen that the classification error rate, as well as the number of selected features are taken into consideration and that the problem is formulated as minimization optimization challenge. In this study, α is set to 0.9, while β is adjusted to 0.1.

At the end of a run, the solution with best fitness is determined and results of its evaluation on the testing set were reported. All experiments were conducted in 20 independent runs. All methods, including SOTA metaheuristics used in comparative analysis shown in [22], along with original bSSA and bSSARM-SCA are implemented in Python using numpy, pandas, scikitlearn and matplotlib libraries. Moreover, the same performance metrics as in [22] are shown and for all implemented methods, a V-shaped transfer function is used for mapping continuous to binary search space. Algorithms proposed in [22] were tested with the control parameters as suggested in original papers.

Finally, as proposed in [40], four different initialization methods were employed in order to more objectively evaluate proposed method: small, mixed and large. In small initialization, all individuals are generated at the beginning of a run with small number of selected features (about 1/3) and in the case of a large individuals employ most of the features ([2/3,1]). In mixed initialization experiments, generated solutions take into account about 2/3 of all features in the dataset.

In all three experiments mean fitness and accuracy obtained over 20 runs are used as performance metrics and expressions used for its calculation are given in Equation (9) and Equation (10), respectively.

(9) $$Avg\left(f\right)=\frac{1}{Run}\sum_{i=1}^{Run}f_{i}^{*},$$

where average fitness is denoted as Avg(f), $f^{*}$ designates the individual with the best fitness in the run, while Run represents the total number of runs.

(10) $$Avg\left(c\right)=\frac{1}{Run}\sum_{i=1}^{Run}\frac{1}{N}\sum_{i=1}^{N}Match\left(C_{i},L_{i}\right),$$

where Avg(c) represents the average classification accuracy, *N* marks the number of instances in the test set, $C_{i}$ represents the classifier output for instance *i*, and $L_{i}$ denotes the reference class corresponding to the given instance *i*.

As already noted, for the purpose of comparative analysis, besides original SSA adapted for binary optimization problems (bSSA), the following algorithms, which results are shown in [22], are also used: whale optimization algorithm (WOA) [12], bWOA with sigmoidal transfer function (bWOA-S) and with hyperbolic tangent transfer function (bWOA-V) [22], three versions of binary ant lion optimizer (BALO) [22], particle swarm optimization (PSO) [75], binary gray wolf optimization (bGWO) and binary dragonfly algorithm (bDA) [11].

Mean fitness and classification accuracy for all three initialization strategies and 21 UCI datasets are shown in Table 10, Table 11, Table 12, Table 13, Table 14 and Table 15. In all provided tables, the best results are marked with bold style.

From the provided experimental results, a few important remarks can be deduced. First, similar results for WOA, bWOA-S, bWOA-v, BALO1, BALO2, BALO3, PSO, bGWO and bDA to those reported in [22] were obtained, therefore validity of previous study is confirmed (it is noted that due to stochastic nature of metaheuristics, exactly the same results could not be generated). Second, proposed hybrid bSSARM-SCA for most datasets and benchmark instances outscores original SSA, hence performance improvements over basic implementation are clear. Finally, when compared to all other SOTA approaches encompassed by comparative analysis, proposed bSSARM-SCA in average obtained the best results and proved to be robust method in tackling feature selection challenge in terms of employed fitness function and classification accuracy.

Formulated fitness function takes into account the number of selected features, however only with weighted coefficient of 0.1 (parameter β=0.1 in expression (8)). For that reason, to further validate propose method the average proportion of selected features (selection size) over 20 runs and all three initialization strategies are shown in Table 16.

Similar to results with an average obtained fitness function and classification accuracy, from Table 16 it can be concluded that on average proposed bSSARM-SCA metaheuristics managed to significantly reduce the number of selected features and this in turn has implications for the classifier’s computational efficiency. Therefore, as a conclusion by performing feature selection with bSSARM-SCA classification computational time can be substantially reduced. In terms of average selection size, only the bDA for some test instances managed to outscore the method proposed in this study.

**Figure 3:**
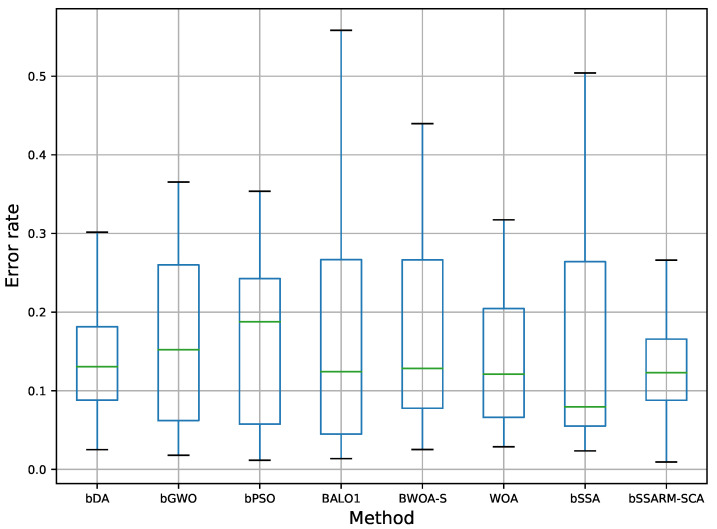
Box plots and whiskers diagrams for average error rate including all datasets and three initialization strategies.

Box and whiskers diagram visualization of average classification error ($E_{R}$) for all datasets and three initialization strategy is shown in Figure 3. From presented diagram stability of propose bSSARM-SCA can be undoubtedly noticed. For example, when compared with basic SSA, that in some runs misses promising regions of the search space, the superiority of the algorithm proposed in this study is evident.

Finally, to show the performances of the proposed bSSARM-SCA algorithm and compare it to other SOTA SSA versions, the authors have implemented binary versions of three novel SSA modifications. The accuracy of the bSSARM-SCA over 21 datasets was compared to opposition based learning and inertia weight ISSA (bISSA1), proposed by [41], opposition based learning and local search ISSA (bISSA2) proposed in [42], and inertia weight ISSA (bISSA3) given in [43]. Again, it is worth noting that the authors have independently implemented all three mentioned binary ISSA variants and executed the experiments with 21 observed datasets. The obtained results are shown in Table 17, where the best result is marked bold for each category (small, large or mixed initialization). The simulation findings clearly show the superiority of the proposed bSSARM-SCA method, that obtained the best results on 15 out of 21 observed datasets. The second best method was bISSA2 [42], which obtained the best results on four datasets, while the bISSA1 method [41] achieved the best accuracy on two datasets.

## 6. Conclusions

Research proposed in this study presents a novel SSA algorithm that addresses observed deficiencies of its original implementation. By hybridizing basic algorithm with well-known SCA metaheuristics and by incorporating guided replacement mechanism, a novel SSARM-SCA metaheurisitcs is devised.

Guided by established practice from the modern literature, before its application to feature selection, the proposed enhanced SSA is firstly tested and evaluated on a recognized test-bed with challenging instances of functions having 30 dimensions from the CEC2013 benchmark suite. Afterwards, it is adapted as a wrapper-based approach for feature selection and validated against 21 well-known datasets retrieved from UCI.

According to experimental findings and rigorous comparative analysis with other recent SOTA approaches, proposed SSARM-SCA proves to be an efficient optimizer that significantly improves convergences speed and results’ quality of the basic SSA and also other SOTA algorithms. Moreover, obtained results prove that the proposed method manage to established better classification accuracy and utilization of lesser number of features, therefore it also manages to improve the solution to the feature selection challenge.

The proposed SSARM-SCA algorithm does not increase the complexity of the basic SSA implementation in terms of FFE, while offering significantly better performances for this particular problem. However, according to the no free lunch theorem, the limitation of the proposed solution is that there are no guarantees that it would perform well for other optimization problems.

The possible directions of the future research include testing of the devised SSARM-SCA algorithm on other practical datasets from different application domains, and also applying it to other optimization problems, such as the wireless sensor networks optimization problem and task scheduling in cloud-based systems.

## Figures and Tables

**Table 1:** CEC2013 benchmark suite details.

No	Functions	Initial Range
Unimodal Functions		
1	Sphere function	${\left[-100,100\right]}^{D}$
2	Rotated High Conditioned Elliptic Function	${\left[-100,100\right]}^{D}$
3	Rotated Bent Cigar Function	${\left[-100,100\right]}^{D}$
4	Rotated Discus Function	${\left[-100,100\right]}^{D}$
5	Different Powers Function	${\left[-100,100\right]}^{D}$
Basic multimodal Functions		
6	Rotated Rosenbrock’s Function	${\left[-100,100\right]}^{D}$
7	Rotated Schaffer’s F7 Function	${\left[-100,100\right]}^{D}$
8	Rotated Ackley’s Function	${\left[-100,100\right]}^{D}$
9	Rotated Weierstrass Function	${\left[-100,100\right]}^{D}$
10	Rotated Griewank’s Function	${\left[-100,100\right]}^{D}$
11	Rastrigin’s Function	${\left[-100,100\right]}^{D}$
12	Rotated Rastrigin’s Function	${\left[-100,100\right]}^{D}$
13	Non-Continuous Rotated Rastrigin’s Function	${\left[-100,100\right]}^{D}$
14	Schwefel’s Function	${\left[-100,100\right]}^{D}$
15	Rotated Schwefel’s Function	${\left[-100,100\right]}^{D}$
16	Rotated Katsuura Function	${\left[-100,100\right]}^{D}$
17	Lunacek Bi_Rastrigin Function	${\left[-100,100\right]}^{D}$
18	Rotated Lunacek Bi_Rastrigin Function	${\left[-100,100\right]}^{D}$
19	Expanded Griewank’s plus Rosenbrock’s Function	${\left[-100,100\right]}^{D}$
20	Expanded Schaffer’s F6 Function	${\left[-100,100\right]}^{D}$
Composition Functions		
21	Composition Function 1 (*n* = 5, Rotated)	${\left[-100,100\right]}^{D}$
22	Composition Function 2 (*n* = 3, Unrotated)	${\left[-100,100\right]}^{D}$
23	Composition Function 3 (*n* = 3, Rotated)	${\left[-100,100\right]}^{D}$
24	Composition Function 4 (*n* = 3, Rotated)	${\left[-100,100\right]}^{D}$
25	Composition Function 5 (*n* = 3, Rotated)	${\left[-100,100\right]}^{D}$
26	Composition Function 6 (*n* = 5, Rotated)	${\left[-100,100\right]}^{D}$
27	Composition Function 7 (*n* = 5, Rotated)	${\left[-100,100\right]}^{D}$
28	Composition Function 8 (*n* = 5, Rotated)	${\left[-100,100\right]}^{D}$

**Table 2:** Comparative analysis between SSARM-SCA and other SOTA methods for CEC2013 unimodal benchmarks.

	SSA	RGA	GSA	D-GSA	BH-GSA	C-GSA	AR-GSA	SSARM-SCA
F1								
Best	$0\times 10^{0}$	$1.85\times 10^{2}$	$0\times 10^{0}$	$6.70\times 10^{-1}$	$4.55\times 10^{-13}$	$2.27\times 10^{-13}$	$0\times 10^{0}$	$0\times 10^{0}$
Median	$0\times 10^{0}$	$2.81\times 10^{2}$	$0\times 10^{0}$	$9.56\times 10^{-1}$	$3.64\times 10^{-12}$	$2.27\times 10^{-13}$	$0\times 10^{0}$	$0\times 10^{0}$
Worst	$2.11\times 10^{-13}$	$3.52\times 10^{2}$	$2.27\times 10^{-13}$	$1.47\times 10^{0}$	$5.00\times 10^{-12}$	$4.55\times 10^{-13}$	$0\times 10^{0}$	$0\times 10^{0}$
Mean	$6.94\times 10^{-14}$	$2.82\times 10^{2}$	$7.58\times 10^{-14}$	$9.75\times 10^{-1}$	$3.33\times 10^{-12}$	$2.76\times 10^{-13}$	$0\times 10^{0}$	$0\times 10^{0}$
Std	$1.11\times 10^{-13}$	$3.16\times 10^{1}$	$1.08\times 10^{-13}$	$1.97\times 10^{-1}$	$1.03\times 10^{-12}$	$9.44\times 10^{-14}$	$0\times 10^{0}$	$0\times 10^{0}$
F2								
Best	$9.16\times 10^{5}$	$1.09\times 10^{7}$	$9.26\times 10^{5}$	$7.26\times 10^{6}$	$5.25\times 10^{5}$	$9.60\times 10^{5}$	$1.56\times 10^{5}$	$1.22\times 10^{5}$
Median	$1.69\times 10^{6}$	$1.59\times 10^{7}$	$1.74\times 10^{6}$	$1.12\times 10^{7}$	$1.97E\times 10^{6}$	$1.75\times 10^{6}$	$6.05\times 10^{5}$	$5.78\times 10^{5}$
Worst	$3.51\times 10^{6}$	$2.53\times 10^{7}$	$3.33\times 10^{6}$	$1.80\times 10^{7}$	$4.94\times 10^{6}$	$3.07\times 10^{6}$	$6.57\times 10^{6}$	$6.48\times 10^{6}$
Mean	$1.21\times 10^{6}$	$1.69\times 10^{7}$	$1.85\times 10^{6}$	$1.16\times 10^{7}$	$2.01\times 10^{6}$	$1.84\times 10^{6}$	$1.37\times 10^{6}$	$1.20\times 10^{6}$
Std	$5.22\times 10^{5}$	$3.64\times 10^{6}$	$5.12\times 10^{5}$	$42.17\times 10^{6}$	$7.84\times 10^{5}$	$4.53\times 10^{5}$	$1.68\times 10^{6}$	$1.45\times 10^{6}$
F3								
Best	$2.65\times 10^{7}$	$3.34\times 10^{9}$	$2.78\times 10^{7}$	$1.02\times 10^{9}$	$4.87\times 10^{-5}$	$2.86\times 10^{7}$	$7.73\times 10^{-12}$	$7.42\times 10^{-12}$
Median	$7.67\times 10^{8}$	$6.28\times 10^{9}$	$7.85\times 10^{8}$	$2.96\times 10^{9}$	$1.62\times 10^{6}$	$1.07\times 10^{9}$	$1.23\times 10^{-11}$	$1.15\times 10^{-11}$
Worst	$2.89\times 10^{9}$	$2.37\times 10^{10}$	$2.95\times 10^{9}$	$9.22\times 10^{9}$	$2.90\times 10^{19}$	$4.38\times 10^{9}$	$1.50\times 10^{-11}$	$1.45\times 10^{-11}$
Mean	$9.71\times 10^{8}$	$6.72\times 10^{9}$	$9.84\times 10^{8}$	$3.55\times 10^{9}$	$5.70\times 10^{17}$	$1.23\times 10^{9}$	$1.19\times 10^{-11}$	$1.07\times 10^{-11}$
Std	$7.23\times 10^{8}$	$2.99\times 10^{9}$	$7.14\times 10^{8}$	$1.72\times 10^{9}$	$4.07\times 10^{18}$	$8.40\times 10^{8}$	$1.85\times 10^{-12}$	$1.76\times 10^{-12}$
F4								
Best	$5.64\times 10^{4}$	$5.16\times 10^{4}$	$5.73\times 10^{4}$	$5.85\times 10^{4}$	$4.99\times 10^{4}$	$5.59\times 10^{4}$	$4.64\times 10^{4}$	$4.43\times 10^{4}$
Median	$6.57\times 10^{4}$	$7.12\times 10^{4}$	$6.86\times 10^{4}$	$6.87\times 10^{4}$	$6.82\times 10^{4}$	$6.98\times 10^{4}$	$6.49\times 10^{4}$	$6.23\times 10^{4}$
Worst	$7.81\times 10^{4}$	$1.03\times 10^{5}$	$7.95\times 10^{4}$	$7.10\times 10^{4}$	$9.03\times 10^{4}$	$8.64\times 10^{4}$	$7.84\times 10^{4}$	$7.66\times 10^{4}$
Mean	$6.72\times 10^{4}$	$7.33\times 10^{4}$	$6.85\times 10^{4}$	$6.74\times 10^{4}$	$6.85\times 10^{4}$	$7.06\times 10^{4}$	$6.47\times 10^{4}$	$6.23\times 10^{4}$
Std	$5.56\times 10^{3}$	$1.20\times 10^{4}$	$5.67\times 10^{3}$	$3.36\times 10^{3}$	$8.16\times 10^{3}$	$5.19\times 10^{3}$	$7.86\times 10^{3}$	$7.54\times 10^{3}$
F5								
Best	$1.56\times 10^{-12}$	$1.95\times 10^{2}$	$1.48\times 10^{-12}$	$2.68\times 10^{0}$	$1.92\times 10^{-11}$	$1.44\times 10^{-11}$	$2.03\times 10^{-8}$	$2.36\times 10^{-8}$
Median	$2.51\times 10^{-12}$	$3.04\times 10^{2}$	$2.39\times 10^{-12}$	$1.49\times 10^{1}$	$1.00\times 10^{-10}$	$2.13\times 10^{-11}$	$9.96\times 10^{-8}$	$8.92\times 10^{-8}$
Worst	$3.88\times 10^{-12}$	$4.65\times 10^{2}$	$3.75\times 10^{-12}$	$6.05\times 10^{1}$	$3.37\times 10^{-10}$	$5.71\times 10^{-11}$	$1.87\times 10^{-7}$	$2.94\times 10^{-7}$
Mean	$2.49\times 10^{-12}$	$3.05\times 10^{2}$	$2.40\times 10^{-12}$	$1.87\times 10^{1}$	$1.25\times 10^{-10}$	$2.36\times 10^{-11}$	$1.02\times 10^{-7}$	$1.58\times 10^{-7}$
Std	$5.67\times 10^{-13}$	$6.13\times 10^{1}$	$5.42\times 10^{-13}$	$1.10\times 10^{1}$	$7.15\times 10^{-11}$	$7.49\times 10^{-12}$	$3.59\times 10^{-8}$	$3.61\times 10^{-8}$

**Table 3:** Comparative analysis between SSARM-SCA and other SOTA methods for CEC2013 multimodal benchmarks.

	SSA	RGA	GSA	D-GSA	BH-GSA	C-GSA	AR-GSA	SSARM-SCA
F6								
Best	$2.56\times 10^{-1}$	$7.81\times 10^{1}$	$2.47\times 10^{-1}$	$5.61\times 10^{-1}$	$2.23\times 10^{-1}$	$1.46\times 10^{-1}$	$3.74\times 10^{-1}$	$2.95\times 10^{-1}$
Median	$5.54\times 10^{1}$	$1.11\times 10^{2}$	$5.69\times 10^{1}$	$7.19\times 10^{1}$	$3.35\times 10^{0}$	$5.43\times 10^{1}$	$1.77\times 10^{1}$	$1.56\times 10^{1}$
Worst	$9.35\times 10^{1}$	$1.39\times 10^{2}$	$9.42\times 10^{1}$	$1.35\times 10^{2}$	$6.86\times 10^{1}$	$1.04\times 10^{2}$	$8.11\times 10^{1}$	$7.87\times 10^{1}$
Mean	$5.24\times 10^{1}$	$1.13\times 10^{2}$	$5.18\times 10^{1}$	$7.36\times 10^{1}$	$2.26\times 10^{1}$	$5.13\times 10^{1}$	$3.37\times 10^{1}$	$3.15\times 10^{1}$
Std	$2.67\times 10^{1}$	$1.20\times 10^{1}$	$2.51\times 10^{1}$	$2.46\times 10^{1}$	$2.68\times 10^{1}$	$2.50\times 10^{1}$	$2.73\times 10^{1}$	$2.58\times 10^{1}$
F7								
Best	$2.69\times 10^{1}$	$4.14\times 10^{1}$	$2.74\times 10^{1}$	$3.61\times 10^{1}$	$4.50\times 10^{-5}$	$3.06\times 10^{1}$	$4.32\times 10^{-9}$	$4.19\times 10^{-9}$
Median	$4.57\times 10^{1}$	$5.57\times 10^{1}$	$4.45\times 10^{1}$	$5.59\times 10^{1}$	$5.23\times 10^{-1}$	$4.35\times 10^{1}$	$2.58\times 10^{-5}$	$2.21\times 10^{-5}$
Worst	$8.56\times 10^{1}$	$6.87\times 10^{1}$	$8.48\times 10^{1}$	$9.11\times 10^{1}$	$2.86\times 10^{1}$	$7.39\times 10^{1}$	$3.63\times 10^{-3}$	$3.15\times 10^{-3}$
Mean	$4.93\times 10^{1}$	$5.58\times 10^{1}$	$4.71\times 10^{1}$	$5.70\times 10^{1}$	$5.59\times 10^{1}$	$4.62\times 10^{1}$	$1.55\times 10^{-4}$	$1.12\times 10^{-4}$
Std	$1.35\times 10^{1}$	$5.66\times 10^{0}$	$1.19\times 10^{1}$	$1.25\times 10^{1}$	$7.62\times 10^{1}$	$1.08\times 10^{1}$	$5.20\times 10^{-4}$	$5.01\times 10^{-4}$
F8								
Best	$2.12\times 10^{1}$	$2.08\times 10^{1}$	$2.08\times 10^{1}$	$2.08\times 10^{1}$	$2.08\times 10^{1}$	$2.09\times 10^{1}$	$2.06\times 10^{1}$	$1.79\times 10^{1}$
Median	$2.15\times 10^{1}$	$2.10\times 10^{1}$	$2.10\times 10^{1}$	$2.10\times 10^{1}$	$2.10\times 10^{1}$	$2.12\times 10^{1}$	$2.10\times 10^{1}$	$1.96\times 10^{1}$
Worst	$2.26\times 10^{1}$	$2.10\times 10^{1}$	$2.10\times 10^{1}$	$2.11\times 10^{1}$	$2.10\times 10^{1}$	$2.16\times 10^{1}$	$2.10\times 10^{1}$	$2.04\times 10^{1}$
Mean	$2.17\times 10^{1}$	$2.10\times 10^{1}$	$2.10\times 10^{1}$	$2.10\times 10^{1}$	$2.10\times 10^{1}$	$2.12\times 10^{1}$	$2.09\times 10^{1}$	$1.85\times 10^{1}$
Std	$4.64\times 10^{-2}$	$4.67\times 10^{-2}$	$4.79\times 10^{-2}$	$5.29\times 10^{-2}$	$5.62\times 10^{-2}$	$1.59\times 10^{-1}$	$7.14\times 10^{-2}$	$4.54\times 10^{-2}$
F9								
Best	$2.36\times 10^{1}$	$1.60\times 10^{1}$	$2.14\times 10^{1}$	$2.11\times 10^{1}$	$3.24\times 10^{0}$	$2.02\times 10^{1}$	$2.37\times 10^{-7}$	$2.24\times 10^{-7}$
Median	$2.97\times 10^{1}$	$2.13\times 10^{1}$	$2.73\times 10^{1}$	$3.01\times 10^{1}$	$7.20\times 10^{1}$	$2.86\times 10^{1}$	$4.99\times 10^{0}$	$4.67\times 10^{0}$
Worst	$3.89\times 10^{1}$	$2.67\times 10^{1}$	$3.50\times 10^{1}$	$3.76\times 10^{1}$	$1.50\times 10^{1}$	$3.68\times 10^{1}$	$8.91\times 10^{0}$	$8.84\times 10^{0}$
Mean	$2.86\times 10^{1}$	$2.16\times 10^{1}$	$2.77\times 10^{1}$	$3.01\times 10^{1}$	$7.78\times 10^{0}$	$2.83\times 10^{1}$	$5.25\times 10^{0}$	$5.13\times 10^{0}$
Std	$3.64\times 10^{0}$	$2.35\times 10^{0}$	$3.56\times 10^{0}$	$3.92\times 10^{0}$	$2.44\times 10^{0}$	$3.65\times 10^{0}$	$1.98\times 10^{0}$	$1.86\times 10^{0}$
F10								
Best	$0\times 10^{0}$	$3.54\times 10^{1}$	$0\times 10^{0}$	$1.22\times 10^{0}$	$5.68\times 10^{-13}$	$3.41\times 10^{-13}$	$0\times 10^{0}$	$0\times 10^{0}$
Median	$5.44\times 10^{-14}$	$5.95\times 10^{1}$	$5.68\times 10^{-14}$	$1.49\times 10^{0}$	$1.19\times 10^{-12}$	$7.40\times 10^{-3}$	$0\times 10^{0}$	$0\times 10^{0}$
Worst	$2.17\times 10^{-2}$	$6.98\times 10^{1}$	$2.22\times 10^{-2}$	$2.20\times 10^{0}$	$1.72\times 10^{-2}$	$2.96\times 10^{-2}$	$1.48\times 10^{-2}$	$1.36\times 10^{-2}$
Mean	$5.55\times 10^{-3}$	$5.91\times 10^{1}$	$5.61\times 10^{-3}$	$1.57\times 10^{0}$	$2.56\times 10^{-3}$	$7.39\times 10^{-3}$	$1.69\times 10^{-3}$	$1.53\times 10^{-3}$
Std	$6.41\times 10^{-3}$	$6.75\times 10^{0}$	$6.39\times 10^{-3}$	$2.68\times 10^{-1}$	$5.05\times 10^{-3}$	$6.02\times 10^{-3}$	$3.83\times 10^{-3}$	$3.67\times 10^{-3}$
F11								
Best	$1.28\times 10^{2}$	$1.14\times 10^{2}$	$1.33\times 10^{2}$	$1.30\times 10^{2}$	$8.95\times 10^{0}$	$1.43\times 10^{2}$	$7.96\times 10^{0}$	$7.76\times 10^{0}$
Median	$1.79\times 10^{2}$	$1.45\times 10^{2}$	$1.83\times 10^{2}$	$1.85\times 10^{2}$	$1.69\times 10^{1}$	$1.84\times 10^{2}$	$1.79\times 10^{1}$	$1.85\times 10^{1}$
Worst	$2.46\times 10^{2}$	$1.62\times 10^{2}$	$2.34\times 10^{2}$	$2.31\times 10^{2}$	$3.38\times 10^{1}$	$2.34\times 10^{2}$	$2.98\times 10^{1}$	$2.75\times 10^{1}$
Mean	$1.95\times 10^{2}$	$1.44\times 10^{2}$	$1.90\times 10^{2}$	$1.87\times 10^{2}$	$1.79\times 10^{1}$	$1.87\times 10^{2}$	$1.83\times 10^{1}$	$1.74\times 10^{1}$
Std	$2.41\times 10^{1}$	$9.16\times 10^{0}$	$2.35\times 10^{1}$	$2.18\times 10^{1}$	$5.21\times 10^{0}$	$2.14\times 10^{1}$	$4.47\times 10^{0}$	$4.32\times 10^{0}$
F12								
Best	$1.67\times 10^{2}$	$1.42\times 10^{2}$	$1.60\times 10^{2}$	$1.52\times 10^{2}$	$7.96\times 10^{0}$	$1.47\times 10^{2}$	$1.29\times 10^{1}$	$1.12\times 10^{1}$
Median	$2.12\times 10^{2}$	$1.58\times 10^{2}$	$2.08\times 10^{2}$	$2.09\times 10^{2}$	$1.39\times 10^{1}$	$2.05\times 10^{2}$	$2.29\times 10^{1}$	$2.12\times 10^{1}$
Worst	$2.68\times 10^{2}$	$1.75\times 10^{2}$	$2.59\times 10^{2}$	$2.63\times 10^{2}$	$2.49\times 10^{1}$	$2.62\times 10^{2}$	$3.88\times 10^{1}$	$3.64\times 10^{1}$
Mean	$2.18\times 10^{2}$	$1.58\times 10^{2}$	$2.07\times 10^{2}$	$2.08\times 10^{2}$	$1.42\times 10^{1}$	$2.06\times 10^{2}$	$2.36\times 10^{1}$	$2.25\times 10^{1}$
Std	$2.83\times 10^{1}$	$8.64\times 10^{0}$	$2.75\times 10^{1}$	$2.39\times 10^{1}$	$3.77\times 10^{0}$	$2.34\times 10^{1}$	$5.42\times 10^{0}$	$5.23\times 10^{0}$
F13								
Best	$2.47\times 10^{2}$	$1.31\times 10^{2}$	$2.75\times 10^{2}$	$2.50\times 10^{2}$	$5.16\times 10^{0}$	$2.43\times 10^{2}$	$1.15\times 10^{1}$	$4.89\times 10^{0}$
Median	$3.48\times 10^{2}$	$1.58\times 10^{2}$	$3.30\times 10^{2}$	$3.24\times 10^{2}$	$2.51\times 10^{1}$	$3.37\times 10^{2}$	$4.13\times 10^{1}$	$2.15\times 10^{0}$
Worst	$4.56\times 10^{2}$	$1.69\times 10^{2}$	$4.28\times 10^{2}$	$4.27\times 10^{2}$	$6.17\times 10^{1}$	$4.06\times 10^{2}$	$8.74\times 10^{1}$	$6.10\times 10^{1}$
Mean	$3.56\times 10^{2}$	$1.57\times 10^{2}$	$3.34\times 10^{2}$	$3.30\times 10^{2}$	$2.77\times 10^{1}$	$3.32\times 10^{2}$	$4.51\times 10^{1}$	$2.43\times 10^{1}$
Std	$3.45\times 10^{1}$	$7.02\times 10^{0}$	$3.34\times 10^{1}$	$3.79\times 10^{1}$	$1.31\times 10^{1}$	$3.96\times 10^{1}$	$1.83\times 10^{1}$	$6.58\times 10^{0}$
F14								
Best	$2.15\times 10^{3}$	$4.36\times 10^{3}$	$2.22\times 10^{3}$	$2.50\times 10^{3}$	$1.06\times 10^{3}$	$2.20\times 10^{3}$	$7.80\times 10^{2}$	$7.21\times 10^{2}$
Median	$3.44\times 10^{3}$	$5.03\times 10^{3}$	$3.26\times 10^{3}$	$3.40\times 10^{3}$	$1.63\times 10^{3}$	$3.33\times 10^{3}$	$1.47\times 10^{3}$	$1.25\times 10^{3}$
Worst	$4.37\times 10^{3}$	$5.64\times 10^{3}$	$4.30\times 10^{3}$	$4.31\times 10^{3}$	$2.60\times 10^{3}$	$4.61\times 10^{3}$	$2.45\times 10^{3}$	$2.33\times 10^{3}$
Mean	$3.45\times 10^{3}$	$5.06\times 10^{3}$	$3.29\times 10^{3}$	$3.38\times 10^{3}$	$1.63\times 10^{3}$	$3.41\times 10^{3}$	$1.49\times 10^{3}$	$1.34\times 10^{3}$
Std	$4.88\times 10^{2}$	$2.62\times 10^{2}$	$4.98\times 10^{2}$	$4.19\times 10^{2}$	$3.24\times 10^{2}$	$4.87\times 10^{2}$	$3.76\times 10^{2}$	$2.59\times 10^{2}$
F15								
Best	$2.45\times 10^{3}$	$4.56\times 10^{3}$	$2.39\times 10^{3}$	$2.14\times 10^{3}$	$5.12\times 10^{2}$	$2.28\times 10^{3}$	$5.33\times 10^{2}$	$5.01\times 10^{2}$
Median	$3.39\times 10^{3}$	$5.30\times 10^{3}$	$3.27\times 10^{3}$	$3.36\times 10^{3}$	$1.19\times 10^{3}$	$3.20\times 10^{3}$	$1.20\times 10^{3}$	$1.04\times 10^{3}$
Worst	$4.85\times 10^{3}$	$5.94\times 10^{3}$	$4.68\times 10^{3}$	$4.99\times 10^{3}$	$2.27\times 10^{3}$	$4.10\times 10^{3}$	$1.82\times 10^{3}$	$2.35\times 10^{3}$
Mean	$3.48\times 10^{3}$	$5.31\times 10^{3}$	$3.31\times 10^{3}$	$3.42\times 10^{3}$	$1.22\times 10^{3}$	$3.28\times 10^{3}$	$1.21\times 10^{3}$	$1.15\times 10^{3}$
Std	$5.65\times 10^{2}$	$2.91\times 10^{2}$	$5.43\times 10^{2}$	$4.92\times 10^{2}$	$3.90\times 10^{2}$	$4.56\times 10^{2}$	$3.29\times 10^{2}$	$2.78\times 10^{2}$
F16								
Best	$4.23\times 10^{-4}$	$1.93\times 10^{0}$	$4.07\times 10^{-4}$	$6.99\times 10^{-1}$	$6.07\times 10^{-4}$	$6.07\times 10^{-4}$	$5.47\times 10^{-4}$	$5.29\times 10^{-4}$
Median	$2.22\times 10^{-3}$	$2.50\times 10^{0}$	$2.11\times 10^{-3}$	$1.13\times 10^{0}$	$3.33\times 10^{-3}$	$2.56\times 10^{-3}$	$2.06\times 10^{-3}$	$2.25\times 10^{-3}$
Worst	$9.54\times 10^{-3}$	$3.02\times 10^{0}$	$9.39\times 10^{-3}$	$1.73\times 10^{0}$	$1.16\times 10^{-2}$	$9.32\times 10^{-3}$	$1.04\times 10^{-2}$	$1.36\times 10^{-2}$
Mean	$2.76\times 10^{-3}$	$2.46\times 10^{0}$	$2.87\times 10^{-3}$	$1.14\times 10^{-3}$	$4.00\times 10^{-3}$	$3.43\times 10^{-3}$	$2.72\times 10^{-3}$	$2.58\times 10^{-3}$
Std	$2.34\times 10^{-3}$	$2.75\times 10^{-1}$	$2.17\times 10^{-3}$	$2.27\times 10^{-1}$	$2.29\times 10^{-3}$	$2.25\times 10^{-3}$	$1.84\times 10^{-3}$	$1.34\times 10^{-3}$
F17								
Best	$3.66\times 10^{1}$	$1.92\times 10^{2}$	$3.74\times 10^{1}$	$7.46\times 10^{1}$	$3.69\times 10^{1}$	$3.58\times 10^{1}$	$4.10\times 10^{1}$	$3.46\times 10^{1}$
Median	$4.25\times 10^{1}$	$2.11\times 10^{2}$	$4.43\times 10^{1}$	$1.04\times 10^{2}$	$4.62\times 10^{1}$	$4.33\times 10^{1}$	$5.04\times 10^{1}$	$4.68\times 10^{1}$
Worst	$6.41\times 10^{1}$	$2.30\times 10^{2}$	$6.67\times 10^{1}$	$1.25\times 10^{2}$	$5.63\times 10^{1}$	$5.68\times 10^{1}$	$6.53\times 10^{1}$	$7.83\times 10^{1}$
Mean	$4.62\times 10^{1}$	$2.11\times 10^{2}$	$4.50\times 10^{1}$	$1.02\times 10^{2}$	$4.61\times 10^{1}$	$4.41\times 10^{1}$	$5.05\times 10^{1}$	$4.58\times 10^{1}$
Std	$5.23\times 10^{0}$	$9.40\times 10^{0}$	$5.06\times 10^{0}$	$1.08\times 10^{1}$	$4.12\times 10^{0}$	$4.37\times 10^{0}$	$5.31\times 10^{0}$	$4.06\times 10^{0}$
F18								
Best	$3.75\times 10^{1}$	$1.85\times 10^{2}$	$3.67\times 10^{1}$	$1.33\times 10^{2}$	$3.93\times 10^{1}$	$3.76\times 10^{1}$	$4.16\times 10^{1}$	$3.58\times 10^{1}$
Median	$4.64\times 10^{1}$	$2.09\times 10^{2}$	$4.53\times 10^{1}$	$1.73\times 10^{2}$	$4.69\times 10^{1}$	$4.45\times 10^{1}$	$5.51\times 10^{1}$	$4.47\times 10^{1}$
Worst	$5.86\times 10^{1}$	$2.28\times 10^{2}$	$5.35\times 10^{1}$	$1.97\times 10^{2}$	$5.89\times 10^{1}$	$5.85\times 10^{1}$	$7.12\times 10^{1}$	$5.16\times 10^{1}$
Mean	$4.76\times 10^{1}$	$2.10\times 10^{2}$	$4.52\times 10^{1}$	$1.73\times 10^{2}$	$4.74\times 10^{1}$	$4.56\times 10^{1}$	$5.61\times 10^{1}$	$4.48\times 10^{1}$
Std	$3.84\times 10^{0}$	$8.88\times 10^{0}$	$3.77\times 10^{0}$	$1.39\times 10^{1}$	$4.05\times 10^{0}$	$4.25\times 10^{0}$	$7.12\times 10^{0}$	$3.65\times 10^{0}$
F19								
Best	$1.69\times 10^{0}$	$2.16\times 10^{1}$	$1.78\times 10^{0}$	$4.34\times 10^{0}$	$2.76\times 10^{0}$	$1.76\times 10^{0}$	$2.54\times 10^{0}$	$2.35\times 10^{0}$
Median	$2.61\times 10^{0}$	$2.55\times 10^{1}$	$2.77\times 10^{0}$	$6.47\times 10^{0}$	$4.58\times 10^{0}$	$3.02\times 10^{0}$	$3.54\times 10^{0}$	$3.67\times 10^{0}$
Worst	$4.01\times 10^{0}$	$2.91\times 10^{1}$	$4.40\times 10^{0}$	$1.54\times 10^{1}$	$6.24\times 10^{0}$	$4.41\times 10^{0}$	$6.87\times 10^{0}$	$6.94\times 10^{0}$
Mean	$2.76\times 10^{0}$	$2.54\times 10^{1}$	$2.95\times 10^{0}$	$7.24\times 10^{0}$	$4.70\times 10^{0}$	$3.03\times 10^{0}$	$3.83\times 10^{0}$	$3.94\times 10^{0}$
Std	$6.76\times 10^{-1}$	$1.60\times 10^{0}$	$6.80\times 10^{-1}$	$2.73\times 10^{0}$	$9.52\times 10^{-1}$	$6.28\times 10^{-1}$	$8.88\times 10^{-1}$	$8.53\times 10^{-1}$
F20								
Best	$1.61\times 10^{1}$	$1.50\times 10^{1}$	$1.41\times 10^{1}$	$1.41\times 10^{1}$	$1.50\times 10^{1}$	$1.50\times 10^{1}$	$1.49\times 10^{1}$	$1.46\times 10^{1}$
Median	$1.65\times 10^{1}$	$1.50\times 10^{1}$	$1.50\times 10^{1}$	$1.50\times 10^{1}$	$1.50\times 10^{1}$	$1.50\times 10^{1}$	$1.50\times 10^{1}$	$1.50\times 10^{1}$
Worst	$1.65\times 10^{1}$	$1.50\times 10^{1}$	$1.50\times 10^{1}$	$1.50\times 10^{1}$	$1.50\times 10^{1}$	$1.50\times 10^{1}$	$1.50\times 10^{1}$	$1.50\times 10^{1}$
Mean	$1.65\times 10^{1}$	$1.50\times 10^{1}$	$1.50\times 10^{1}$	$1.50\times 10^{1}$	$1.50\times 10^{1}$	$1.50\times 10^{1}$	$1.50\times 10^{1}$	$1.50\times 10^{1}$
Std	$1.45\times 10^{-1}$	$9.93\times 10^{-6}$	$1.33\times 10^{-1}$	$1.81\times 10^{-1}$	$6.30\times 10^{-8}$	$3.09\times 10^{-6}$	$1.98\times 10^{-2}$	$6.17\times 10^{-8}$

**Table 4:** Comparative analysis between SSARM-SCA and other SOTA methods for CEC2013 composite benchmarks.

	SSA	RGA	GSA	D-GSA	BH-GSA	C-GSA	AR-GSA	SSARM-SCA
F21								
Best	$1.27\times 10^{2}$	$4.62\times 10^{2}$	$1.00\times 10^{2}$	$1.27\times 10^{2}$	$2.00\times 10^{2}$	$1.00\times 10^{2}$	$2.00\times 10^{2}$	$1.95\times 10^{2}$
Median	$3.65\times 10^{2}$	$5.62\times 10^{2}$	$3.00\times 10^{2}$	$3.15\times 10^{2}$	$3.00\times 10^{2}$	$3.00\times 10^{2}$	$3.00\times 10^{2}$	$2.84\times 10^{2}$
Worst	$4.76\times 10^{2}$	$6.07\times 10^{2}$	$4.44\times 10^{2}$	$4.44\times 10^{2}$	$4.44\times 10^{2}$	$4.44\times 10^{2}$	$4.44\times 10^{2}$	$4.26\times 10^{2}$
Mean	$3.36\times 10^{2}$	$5.41\times 10^{2}$	$3.20\times 10^{2}$	$3.40\times 10^{2}$	$3.36\times 10^{2}$	$3.32\times 10^{2}$	$3.26\times 10^{2}$	$3.12\times 10^{2}$
Std	$7.39\times 10^{1}$	$4.30\times 10^{1}$	$7.28\times 10^{1}$	$7.13\times 10^{1}$	$9.12\times 10^{1}$	$7.97\times 10^{1}$	$9.22\times 10^{1}$	$4.26\times 10^{1}$
F22								
Best	$3.95\times 10^{3}$	$4.31\times 10^{3}$	$3.78\times 10^{3}$	$4.03\times 10^{3}$	$3.28\times 10^{2}$	$3.87\times 10^{3}$	$3.13\times 10^{2}$	$3.05\times 10^{2}$
Median	$5.39\times 10^{3}$	$4.99\times 10^{3}$	$5.18\times 10^{3}$	$5.39\times 10^{3}$	$1.10\times 10^{3}$	$5.53\times 10^{3}$	$1.11\times 10^{3}$	$1.05\times 10^{3}$
Worst	$7.25\times 10^{3}$	$5.75\times 10^{3}$	$7.08\times 10^{3}$	$7.04\times 10^{3}$	$2.18\times 10^{3}$	$7.50\times 10^{3}$	$2.26\times 10^{3}$	$2.15\times 10^{3}$
Mean	$5.63\times 10^{3}$	$5.06\times 10^{3}$	$5.35\times 10^{3}$	$5.53\times 10^{3}$	$1.22\times 10^{3}$	$5.51\times 10^{3}$	$1.12\times 10^{3}$	$1.03\times 10^{3}$
Std	$8.91\times 10^{2}$	$3.43\times 10^{2}$	$8.59\times 10^{2}$	$7.93\times 10^{2}$	$4.09\times 10^{2}$	$8.06\times 10^{2}$	$3.83\times 10^{2}$	$3.21\times 10^{2}$
F23								
Best	$4.32\times 10^{3}$	$4.37\times 10^{3}$	$4.23\times 10^{3}$	$4.86\times 10^{3}$	$6.01\times 10^{2}$	$3.86\times 10^{3}$	$1.01\times 10^{3}$	$1.28\times 10^{3}$
Median	$5.67\times 10^{3}$	$5.41\times 10^{3}$	$5.50\times 10^{3}$	$5.54\times 10^{3}$	$1.96\times 10^{3}$	$5.49\times 10^{3}$	$1.84\times 10^{3}$	$1.92\times 10^{3}$
Worst	$6.89\times 10^{3}$	$6.24\times 10^{3}$	$6.67\times 10^{3}$	$6.38\times 10^{3}$	$4.23\times 10^{3}$	$6.12\times 10^{3}$	$3.75\times 10^{3}$	$3.85\times 10^{3}$
Mean	$5.76\times 10^{3}$	$5.40\times 10^{3}$	$5.53\times 10^{3}$	$5.58\times 10^{3}$	$2.10\times 10^{3}$	$5.44\times 10^{3}$	$1.96\times 10^{3}$	$2.10\times 10^{3}$
Std	$4.59\times 10^{2}$	$4.05\times 10^{2}$	$4.36\times 10^{2}$	$3.22\times 10^{2}$	$7.58\times 10^{2}$	$4.30\times 10^{2}$	$6.01\times 10^{2}$	$3.13\times 10^{2}$
F24								
Best	$2.46\times 10^{2}$	$2.31\times 10^{2}$	$2.20\times 10^{2}$	$2.16\times 10^{2}$	$2.00\times 10^{2}$	$2.29\times 10^{2}$	$2.00\times 10^{2}$	$1.96\times 10^{2}$
Median	$2.68\times 10^{2}$	$2.37\times 10^{2}$	$2.57\times 10^{2}$	$2.59\times 10^{2}$	$2.00\times 10^{2}$	$2.55\times 10^{2}$	$2.00\times 10^{2}$	$1.99\times 10^{2}$
Worst	$3.95\times 10^{2}$	$2.80\times 10^{2}$	$3.90\times 10^{2}$	$3.82\times 10^{2}$	$2.10\times 10^{2}$	$3.87\times 10^{2}$	$2.00\times 10^{2}$	$2.07\times 10^{2}$
Mean	$2.81\times 10^{2}$	$2.40\times 10^{2}$	$2.79\times 10^{2}$	$2.71\times 10^{2}$	$2.01\times 10^{2}$	$2.68\times 10^{2}$	$2.00\times 10^{2}$	$2.00\times 10^{0}$
Std	$4.60\times 10^{1}$	$1.12\times 10^{1}$	$4.49\times 10^{1}$	$3.77\times 10^{1}$	$1.18\times 10^{-1}$	$3.63\times 10^{1}$	$2.45\times 10^{-2}$	$2.17\times 10^{-2}$
F25								
Best	$2.22\times 10^{2}$	$2.40\times 10^{2}$	$2.00\times 10^{2}$	$2.09\times 10^{2}$	$2.00\times 10^{2}$	$2.00\times 10^{2}$	$2.00\times 10^{2}$	$1.89\times 10^{2}$
Median	$3.57\times 10^{2}$	$2.83\times 10^{2}$	$3.43\times 10^{2}$	$3.50\times 10^{2}$	$2.00\times 10^{2}$	$3.41\times 10^{2}$	$2.00\times 10^{2}$	$1.95\times 10^{2}$
Worst	$4.11\times 10^{2}$	$3.04\times 10^{2}$	$3.86\times 10^{2}$	$3.86\times 10^{2}$	$2.71\times 10^{2}$	$3.85\times 10^{2}$	$2.00\times 10^{2}$	$1.99\times 10^{2}$
Mean	$3.85\times 10^{2}$	$2.72\times 10^{2}$	$3.32\times 10^{2}$	$3.39\times 10^{2}$	$2.12\times 10^{2}$	$3.32\times 10^{2}$	$2.00\times 10^{2}$	$1.94\times 10^{2}$
Std	$4.55\times 10^{1}$	$2.51\times 10^{1}$	$4.06\times 10^{1}$	$3.71\times 10^{1}$	$2.55\times 10^{1}$	$4.15\times 10^{1}$	$1.86\times 10^{-5}$	$1.82\times 10^{-5}$
F26								
Best	$2.56\times 10^{2}$	$2.01\times 10^{2}$	$2.34\times 10^{2}$	$2.00\times 10^{2}$	$1.11\times 10^{2}$	$2.00\times 10^{2}$	$2.27\times 10^{2}$	$2.31\times 10^{2}$
Median	$3.67\times 10^{2}$	$3.40\times 10^{2}$	$3.42\times 10^{2}$	$3.50\times 10^{2}$	$3.00\times 10^{2}$	$3.48\times 10^{2}$	$2.98\times 10^{2}$	$3.06\times 10^{2}$
Worst	$3.87\times 10^{2}$	$3.64\times 10^{2}$	$3.78\times 10^{2}$	$3.70\times 10^{2}$	$3.25\times 10^{2}$	$3.71\times 10^{2}$	$3.19\times 10^{2}$	$3.25\times 10^{2}$
Mean	$3.43\times 10^{2}$	$3.15\times 10^{2}$	$3.29\times 10^{2}$	$3.33\times 10^{2}$	$2.85\times 10^{2}$	$3.28\times 10^{2}$	$2.92\times 10^{2}$	$2.99\times 10^{2}$
Std	$3.88\times 10^{1}$	$6.06\times 10^{1}$	$3.74\times 10^{1}$	$4.35\times 10^{1}$	$4.26\times 10^{1}$	$4.76\times 10^{1}$	$1.71\times 10^{1}$	$1.68\times 10^{1}$
F27								
Best	$5.64\times 10^{2}$	$6.15\times 10^{2}$	$5.88\times 10^{2}$	$6.11\times 10^{2}$	$3.00\times 10^{2}$	$6.24\times 10^{2}$	$3.00\times 10^{2}$	$3.24\times 10^{2}$
Median	$7.51\times 10^{2}$	$7.96\times 10^{2}$	$7.63\times 10^{2}$	$8.43\times 10^{2}$	$3.00\times 10^{2}$	$7.68\times 10^{2}$	$3.00\times 10^{2}$	$3.27\times 10^{2}$
Worst	$9.76\times 10^{2}$	$1.02\times 10^{3}$	$9.87\times 10^{2}$	$1.03\times 10^{3}$	$3.04\times 10^{2}$	$1.01\times 10^{3}$	$3.03\times 10^{2}$	$3.30\times 10^{2}$
Mean	$7.78\times 10^{2}$	$7.74\times 10^{2}$	$7.84\times 10^{2}$	$8.41\times 10^{2}$	$3.01\times 10^{2}$	$7.84\times 10^{2}$	$3.00\times 10^{2}$	$3.25\times 10^{2}$
Std	$1.02\times 10^{2}$	$1.33\times 10^{2}$	$1.09\times 10^{2}$	$1.12\times 10^{2}$	$1.14\times 10^{0}$	$9.29\times 10^{1}$	$4.09\times 10^{-1}$	$4.34\times 10^{-1}$
F28								
Best	$2.24\times 10^{3}$	$5.09\times 10^{2}$	$2.46\times 10^{3}$	$2.83\times 10^{3}$	$1.00\times 10^{2}$	$2.33\times 10^{3}$	$3.00\times 10^{2}$	$3.23\times 10^{2}$
Median	$3.15\times 10^{3}$	$8.12\times 10^{2}$	$3.13\times 10^{3}$	$3.22\times 10^{3}$	$3.00\times 10^{2}$	$3.17\times 10^{3}$	$3.00\times 10^{2}$	$3.28\times 10^{2}$
Worst	$3.52\times 10^{3}$	$1.75\times 10^{3}$	$3.68\times 10^{3}$	$3.94\times 10^{3}$	$1.35\times 10^{3}$	$3.93\times 10^{3}$	$3.00\times 10^{2}$	$3.34\times 10^{2}$
Mean	$3.05\times 10^{3}$	$8.91\times 10^{2}$	$3.14\times 10^{3}$	$3.25\times 10^{3}$	$3.49\times 10^{2}$	$3.24\times 10^{3}$	$3.00\times 10^{2}$	$3.27\times 10^{2}$
Std	$2.58\times 10^{2}$	$3.52\times 10^{2}$	$2.71\times 10^{2}$	$2.37\times 10^{2}$	$2.53\times 10^{2}$	$2.91\times 10^{2}$	$8.77\times 10^{-9}$	$8.92\times 10^{-9}$

**Table 5:** Friedman test ranks for the compared algorithms over 28 CEC2013 functions.

Functions	SSA	RGA	GSA	D-GSA	BH-GSA	C-GSA	AR-GSA	SSARM-SCA
F1	3	8	4	7	6	5	1.5	1.5
F2	1	8	5	7	6	4	3	2
F3	4	7	3	6	8	5	2	1
F4	6	8	4.5	3	4.5	7	2	1
F5	1	8	2	7	4	3	5	6
F6	4	8	6	7	1	5	3	2
F7	6	7	5	8	3	4	2	1
F8	2	5.5	5.5	5.5	5.5	8	3	1
F9	7	4	5	8	3	6	2	1
F10	5	8	4	7	3	6	2	1
F11	8	4	7	5.5	2	5.5	3	1
F12	5	4	7	8	1	6	3	2
F13	8	4	7	5	2	6	3	1
F14	4	8	5	6	3	7	2	1
F15	4	8	6	7	3	5	2	1
F16	4	8	3	7	6	5	2	1
F17	5	8	2	7	4	1	6	3
F18	5	8	2	7	4	3	6	1
F19	2	8	1	7	6	3	4	5
F20	8	4	4	4	4	4	4	4
F21	1	8	3	7	6	5	4	2
F22	8	4	5	7	3	6	2	1
F23	8	4	6	7	2.5	5	1	2.5
F24	8	4	7	6	3	5	2	1
F25	8	4	5.5	7	3	5.5	2	1
F26	4	5	7	8	1	6	2	3
F27	7	4	5.5	8	2	5.5	1	3
F28	5	4	6	8	3	7	1	2
Average Ranking	5.035714286	6.160714286	4.75	6.678571429	3.660714286	5.125	2.696428571	1.892857143
Rank	5	7	4	8	3	6	2	1

**Table 6:** Aligned Friedman test ranks for the compared algorithms over 28 CEC2013 functions.

Functions	SSA	RGA	GSA	D-GSA	BH-GSA	C-GSA	AR-GSA	SSARM-SCA
F1	64	192	65	68	67	66	62.5	62.5
F2	8	223	12	222	13	11	10	9
F3	4	7	3	6	224	5	1.5	1.5
F4	216	221	195.5	32	195.5	219	15	14
F5	51	194	52	85	54	53	55	56
F6	109	167	115	153	73	112	87	84
F7	148	155	147	157	79	146	71	70
F8	123	132.5	132.5	132.5	132.5	136	130	113
F9	144	139	142	145	95	143	93	92
F10	103	164	102	105	101	104	100	99
F11	175	156	170	168.5	44	168.5	45	43
F12	171	159	173	174	40	172	42	41
F13	190	50	182	179	38	180	39	37
F14	187	218	197	199	30	201	29	27
F15	193	220	200	202	24	198	23	22
F16	127	138	126	137	129	128	125	124
F17	82	183	75	158	77	74	83	76
F18	69	181	59	177	61	60	78	57
F19	107	150	106	135	114	108	110	111
F20	140	119	119	119	119	119	119	119
F21	49	189	72	97	91	89	81	58
F22	217	206	212	214	18	213	17	16
F23	215	203	207	208	20.5	204	19	20.5
F24	176	152	166	163	88	162	86	36
F25	178	94	160.5	165	48	160.5	47	46
F26	98	141	151	154	80	149	90	
F27	188	184	185.5	191	34	185.5	33	35
F28	205	31	209	211	28	210	25	26
Average Ranking	133.4642857	156.0178571	133.4285714	148.4642857	75.625	134.875	61.28571429	56.83928571
Rank	5	8	4	7	3	6	2	1

**Table 7:** Friedman and Iman–Davenport statistical test results summary (α=0.05).

Friedman Value	$\chi^{2}$ Critical Value	*p*-Value	Iman-Davenport Value	*F*-Critical Value
$8.866\times 10^{1}$	$1.407\times 10^{1}$	$1.110\times 10^{-16}$	$2.230\times 10^{1}$	$2.058\times 10^{0}$

**Table 8:** Results of the Holm’s step-down procedure.

Comparison	*p*-Values	Ranking	Alpha = 0.05	Alpha = 0.1	H1	H2
SSARM-SCA vs. D-GSA	$1.33227\times 10^{-13}$	0	0.007142857	0.014285714	TRUE	TRUE
SSARM-SCA vs. RGA	$3.53276\times 10^{-11}$	1	0.008333333	0.016666667	TRUE	TRUE
SSARM-SCA vs. C-GSA	$3.96302\times 10^{-7}$	2	0.01	0.02	TRUE	TRUE
SSARM-SCA vs. SSA	$7.90191\times 10^{-7}$	3	0.0125	0.025	TRUE	TRUE
SSARM-SCA vs. GSA	$6.37484\times 10^{-6}$	4	0.016666667	0.033333333	TRUE	TRUE
SSARM-SCA vs. BH-GSA	0.003462325	5	0.025	0.05	TRUE	TRUE
SSARM-SCA vs. AR-GSA	0.109821937	6	0.05	0.1	FALSE	FALSE

**Table 9:** Experimental setup datasets.

No.	Name	Features	Samples
1	Breastcancer	9	699
2	Tic-tac-toe	9	958
3	Zoo	16	101
4	WineEW	13	178
5	SpectEW	22	267
6	SonarEW	60	208
7	IonosphereEW	34	351
8	HeartEW	13	270
9	CongressEW	16	435
10	KrvskpEW	36	3196
11	WaveformEW	40	5000
12	Exactly	13	1000
13	Exactly 2	13	1000
14	M-of-N	13	1000
15	vote	16	300
16	BreastEW	30	569
17	Semeion	265	1593
18	Clean 1	166	476
19	Clean 2	166	6598
20	Lymphography	18	148
21	PenghungEW	325	73

**Table 10:** Mean fitness statistical metric using small initialization with the 21 utilized datasets.

No.	WOA	bWOA-S	bWOA-v	BALO1	BALO2	BALO3	PSO	bGWO	bDA	bSSA	bSSARM-SCA
1	0.063	0.047	0.052	0.078	0.096	0.089	0.059	0.033	0.033	0.153	**0.025**
2	0.327	0.223	0.315	0.347	0.353	0.335	0.332	0.248	**0.217**	0.275	0.224
3	0.242	0.135	0.222	0.413	0.397	0.417	0.258	0.125	**0.052**	0.675	0.154
4	0.939	0.907	0.936	0.954	0.962	0.953	0.927	0.884	0.879	0.915	**0.836**
5	0.341	0.291	0.341	0.353	0.393	0.374	0.361	0.279	0.252	**0.199**	0.634
6	0.332	0.204	0.313	0.375	0.374	0.368	0.304	0.156	0.181	0.097	**0.063**
7	0.136	0.121	0.134	0.172	0.176	0.185	0.143	**0.097**	0.127	0.173	0.116
8	0.291	0.253	0.276	0.297	0.305	0.286	0.284	0.193	0.163	0.194	**0.129**
9	0.380	0.362	0.378	0.393	0.396	0.394	0.403	0.355	0.336	0.498	**0.289**
10	0.395	0.088	0.376	0.423	0.416	0.418	0.422	0.078	0.057	0.294	**0.033**
11	0.434	0.194	0.438	0.496	0.497	0.516	0.436	0.182	0.184	0.171	**0.149**
12	0.323	0.293	0.335	0.348	0.333	0.335	0.318	0.317	0.203	0.368	**0.133**
13	0.244	0.242	0.237	0.239	0.266	0.241	0.245	0.246	0.238	0.257	**0.222**
14	0.298	0.134	0.298	0.357	0.352	0.356	0.282	0.135	0.071	0.245	**0.033**
15	0.129	0.065	0.142	0.153	0.156	0.175	0.135	0.064	0.051	0.064	**0.047**
16	0.052	0.046	0.057	0.084	0.082	0.084	0.054	0.036	0.032	**0.026**	0.049
17	0.098	0.038	0.096	0.092	0.091	0.097	0.097	**0.024**	0.034	0.187	0.715
18	0.294	0.153	0.297	0.359	0.378	0.366	0.292	0.111	0.146	0.975	**0.099**
19	0.083	0.046	0.086	0.127	0.133	0.135	0.085	**0.036**	0.047	0.386	0.231
20	0.298	0.205	0.274	0.374	0.316	0.378	0.302	0.187	0.168	0.256	**0.135**
21	0.467	0.182	0.445	0.615	0.601	0.607	0.449	0.144	0.173	0.132	**0.112**

**Table 11:** Classification accuracy using small initialization for the 21 utilized datasets.

No.	WOA	bWOA-S	bWOA-v	BALO1	BALO2	BALO3	PSO	bGWO	bDA	bSSA	bSSARM-SCA
1	0.864	0.645	0.741	0.832	0.811	0.844	0.862	0.965	0.752	0.856	**0.974**
2	0.658	**0.783**	0.672	0.590	0.593	0.583	0.621	0.746	0.685	0.694	0.780
3	0.744	0.842	0.777	0.458	0.474	0.449	0.585	**0.868**	0.814	0.621	0.665
4	0.042	0.050	0.028	0.012	0.015	0.013	0.034	0.087	0.032	0.331	**0.343**
5	0.627	0.668	0.609	0.563	0.556	0.554	0.584	0.706	0.644	**0.769**	0.754
6	0.639	0.714	0.657	0.543	0.547	0.546	0.605	0.835	0.697	0.903	**0.915**
7	0.844	0.837	0.835	0.784	0.774	0.763	0.822	**0.893**	0.829	0.867	0.874
8	0.675	0.644	0.633	0.605	0.598	0.609	0.655	0.795	0.657	0.763	**0.831**
9	0.582	0.581	0.586	0.558	0.542	0.572	0.566	0.623	0.581	0.910	**0.938**
10	0.580	0.912	0.608	0.514	0.517	0.513	0.547	**0.918**	0.783	0.917	0.916
11	0.552	0.803	0.553	0.392	0.403	0.399	0.391	0.812	0.742	0.795	**0.830**
12	0.636	0.669	0.612	0.589	0.621	0.618	0.655	0.654	0.645	0.678	**0.712**
13	0.729	0.725	0.708	0.748	0.696	0.702	0.726	0.726	0.714	0.746	**0.764**
14	0.692	0.848	0.849	0.722	0.727	0.703	0.817	0.934	0.873	0.857	**0.953**
15	0.861	0.917	0.834	0.723	0.723	0.703	0.818	0.933	0.879	0.745	**0.935**
16	0.895	0.691	0.725	0.805	0.827	0.834	0.899	0.962	0.785	**0.989**	0.977
17	0.898	0.962	0.896	0.878	0.903	0.909	0.895	**0.971**	0.958	0.897	0.914
18	0.680	0.818	0.677	0.590	0.582	0.587	0.644	0.872	0.795	0.874	**0.891**
19	0.908	0.956	0.908	0.841	0.843	0.848	0.883	**0.961**	0.953	0.885	0.904
20	0.677	0.735	0.654	0.517	0.556	0.524	0.612	0.792	0.708	0.702	**0.878**
21	0.496	0.742	0.491	0.284	0.297	0.301	0.417	0.802	0.722	0.825	**0.894**

**Table 12:** Mean fitness statistical metric using large initialization with the 21 utilized datasets.

No.	WOA	bWOA-S	bWOA-v	BALO1	BALO2	BALO3	PSO	bGWO	bDA	bSSA	bSSARM-SCA
1	0.135	0.128	0.167	0.185	0.148	0.226	0.163	**0.031**	0.039	0.159	0.033
2	0.216	0.209	0.208	0.246	0.244	0.241	0.204	0.212	0.208	0.219	**0.191**
3	0.143	0.134	0.130	0.163	0.125	0.189	0.176	0.102	0.076	0.146	**0.068**
4	0.926	0.927	0.923	0.937	0.939	0.923	0.929	0.905	0.886	0.856	**0.829**
5	0.315	0.317	0.313	0.320	0.328	0.318	0.318	0.304	0.243	0.383	**0.214**
6	0.304	0.287	0.299	0.274	0.295	0.285	0.273	0.258	0.194	0.275	**0.138**
7	0.168	0.164	0.184	0.166	0.178	0.164	0.163	0.155	0.124	**0.094**	0.116
8	0.344	0.333	0.341	0.346	0.354	0.348	0.344	0.288	0.177	0.211	**0.133**
9	0.403	0.408	0.393	0.407	0.405	0.387	0.395	0.373	0.342	0.051	**0.039**
10	0.065	0.077	0.074	0.071	0.076	0.076	0.068	0.062	**0.053**	0.071	0.062
11	0.192	0.198	0.195	0.193	0.199	0.194	0.186	0.189	0.186	0.175	**0.159**
12	0.307	0.308	0.313	0.309	0.307	0.303	0.308	0.307	0.209	0.258	**0.192**
13	0.256	0.250	0.261	0.267	0.261	0.262	0.257	0.255	0.243	0.259	**0.227**
14	0.139	0.134	0.137	0.146	0.131	0.138	0.122	0.124	0.065	0.194	**0.054**
15	0.083	0.094	0.087	0.085	0.092	0.095	0.082	0.087	0.058	0.065	**0.051**
16	0.218	0.223	0.153	0.104	0.154	0.208	0.203	0.046	**0.031**	0.105	0.143
17	0.042	0.042	0.049	0.041	0.049	0.046	0.043	0.033	**0.032**	0.145	0.075
18	0.181	0.181	0.181	0.188	0.198	0.194	0.188	0.178	0.136	0.091	**0.074**
19	0.053	0.050	0.057	0.054	0.055	0.055	0.054	0.043	0.047	0.036	**0.022**
20	0.239	0.235	0.223	0.245	0.230	0.237	0.237	0.222	**0.143**	0.250	0.161
21	0.269	0.244	0.277	0.272	0.261	0.278	0.233	0.229	0.181	0.276	**0.156**

**Table 13:** Classification accuracy using large initialization for the 21 utilized datasets.

No.	WOA	bWOA-S	bWOA-v	BALO1	BALO2	BALO3	PSO	bGWO	bDA	bSSA	bSSARM-SCA
1	0.615	0.617	0.612	0.678	0.694	0.667	0.741	**0.950**	0.781	0.925	0.948
2	0.793	0.798	0.797	0.744	0.736	0.740	0.741	0.761	0.664	0.773	**0.814**
3	0.834	0.830	0.835	0.813	0.843	0.793	0.815	0.897	0.785	0.871	**0.920**
4	0.057	0.056	0.056	0.041	0.053	0.064	0.063	0.081	0.035	0.052	**0.094**
5	0.663	0.677	0.660	0.664	0.660	0.672	0.669	0.680	0.649	0.669	**0.714**
6	0.691	0.702	0.690	0.715	0.695	0.701	0.725	0.748	0.703	0.726	**0.794**
7	0.836	0.832	0.812	0.837	0.827	0.834	0.833	0.853	0.811	**0.871**	0.856
8	0.645	0.654	0.633	0.644	0.632	0.635	0.641	0.693	0.653	0.612	**0.715**
9	0.599	0.587	0.594	0.583	0.585	0.598	0.589	0.629	0.586	0.570	**0.645**
10	0.935	**0.939**	0.935	0.912	0.928	0.924	0.930	0.930	0.771	0.856	0.918
11	0.814	0.803	0.817	0.809	0.805	0.811	0.811	0.819	0.743	0.811	**0.830**
12	0.692	0.683	0.688	0.685	0.684	0.672	0.683	0.685	0.649	0.651	**0.707**
13	0.741	0.744	0.744	0.726	0.721	0.728	0.735	0.737	0.710	0.779	**0.786**
14	0.868	0.865	0.862	0.837	0.830	0.837	0.857	0.866	0.727	0.819	**0.886**
15	0.906	0.906	0.903	0.908	0.909	0.909	0.908	0.918	0.884	0.895	**0.931**
16	0.618	0.616	0.614	0.712	0.692	0.654	0.711	**0.937**	0.769	0.715	0.898
17	0.966	0.967	0.965	0.960	0.961	0.966	0.968	**0.971**	0.952	0.945	0.933
18	0.814	0.810	0.816	0.823	0.805	0.813	0.815	0.839	0.801	0.796	**0.861**
19	0.957	0.951	0.957	0.953	0.956	0.950	0.959	0.950	0.950	0.966	**0.978**
20	0.744	0.753	0.763	0.737	0.749	0.757	0.743	**0.773**	0.714	0.726	0.752
21	0.743	0.757	0.730	0.728	0.743	0.731	0.761	0.771	0.735	0.759	**0.793**

**Table 14:** Mean fitness statistical metric using mixed initialization with the 21 utilized datasets.

No.	WOA	bWOA-S	bWOA-v	BALO1	BALO2	BALO3	PSO	bGWO	bDA	bSSA	bSSARM-SCA
1	0.053	0.050	0.078	0.104	0.098	0.073	0.033	0.037	0.031	0.067	**0.025**
2	0.221	0.206	0.212	0.248	0.253	0.244	0.207	0.216	0.207	0.219	**0.191**
3	0.150	0.144	0.122	0.182	0.147	0.142	0.077	0.090	0.074	0.115	**0.059**
4	0.926	0.928	0.911	0.939	0.939	0.933	0.883	0.902	**0.883**	0.926	0.892
5	0.317	0.303	0.287	0.313	0.325	0.315	**0.240**	0.281	0.256	0.302	0.293
6	0.305	0.282	0.259	0.274	0.294	0.289	0.169	0.231	0.195	0.260	**0.154**
7	0.157	0.151	0.154	0.157	0.163	0.168	**0.115**	0.148	0.125	0.151	0.136
8	0.322	0.304	0.250	0.313	0.326	0.308	0.156	0.236	0.168	0.256	**0.137**
9	0.388	0.389	0.373	0.397	0.395	0.383	**0.333**	0.354	0.342	0.358	0.337
10	0.076	0.078	0.082	0.078	0.071	0.077	0.041	0.063	0.055	**0.028**	0.031
11	0.191	0.195	0.194	0.194	0.197	0.193	0.184	0.185	0.183	0.171	**0.155**
12	0.300	0.307	0.305	0.305	0.309	0.307	**0.155**	0.276	0.222	0.254	0.176
13	0.247	0.241	0.256	0.236	0.246	0.254	0.236	0.245	0.242	0.252	**0.223**
14	0.135	0.134	0.157	0.153	0.156	0.139	0.027	0.114	0.071	0.095	**0.025**
15	0.088	0.080	0.089	0.080	0.093	0.082	**0.048**	0.063	0.057	0.064	0.056
16	0.089	0.059	0.063	0.085	0.080	0.083	0.039	0.056	0.036	0.057	**0.031**
17	0.043	0.047	0.034	0.047	0.045	0.049	0.031	0.035	**0.031**	0.043	0.042
18	0.195	0.182	0.178	0.188	0.197	0.196	**0.133**	0.157	0.143	0.167	0.149
19	0.056	0.053	0.043	0.057	0.056	0.053	0.044	0.046	0.046	0.051	**0.034**
20	0.235	0.234	0.225	0.254	0.245	0.235	0.135	0.217	0.163	0.212	**0.123**
21	0.263	0.240	0.241	0.273	0.260	0.275	0.144	0.211	0.183	0.223	**0.128**

**Table 15:** Classification accuracy using mixed initialization for the 21 utilized datasets.

No.	WOA	bWOA-S	bWOA-v	BALO1	BALO2	BALO3	PSO	bGWO	bDA	bSSA	bSSARM-SCA
1	0.784	0.612	0.624	0.749	0.726	0.723	0.803	0.969	0.788	0.988	**0.995**
2	0.786	0.793	0.785	0.685	0.684	0.687	0.722	0.764	0.678	0.784	**0.826**
3	0.847	0.834	0.827	0.652	0.705	0.684	0.787	0.903	0.773	0.949	**0.987**
4	0.068	0.056	0.057	0.037	0.034	0.035	0.038	**0.088**	0.031	0.052	0.079
5	0.672	0.675	0.664	0.636	0.622	0.620	0.653	**0.703**	0.640	0.635	0.694
6	0.690	0.709	0.708	0.644	0.636	0.642	0.722	0.765	0.706	0.687	**0.789**
7	0.833	0.838	0.836	0.812	0.807	0.803	0.834	0.869	0.828	**0.918**	0.894
8	0.654	0.656	0.654	0.628	0.628	0.625	0.661	0.753	0.651	0.654	**0.795**
9	0.595	0.588	0.593	0.574	0.556	0.579	0.583	**0.636**	0.574	0.587	0.611
10	0.936	0.932	0.913	0.768	0.764	0.756	0.795	0.943	0.752	**0.976**	0.971
11	0.817	0.802	0.803	0.647	0.643	0.645	0.766	0.815	0.748	0.821	**0.851**
12	0.683	0.688	0.697	0.642	0.652	0.643	0.662	**0.708**	0.644	0.695	0.696
13	0.737	0.747	0.738	0.731	0.714	0.702	0.727	0.739	0.716	0.750	**0.776**
14	0.862	0.862	0.834	0.731	0.737	0.742	0.763	0.886	0.725	0.792	**0.910**
15	0.917	0.904	0.903	0.822	0.825	0.824	0.889	**0.931**	0.865	0.854	0.893
16	0.768	0.618	0.610	0.735	0.743	0.726	0.812	0.947	0.761	0.789	**0.956**
17	0.969	0.966	0.963	0.926	0.936	0.928	0.955	**0.975**	0.953	0.909	0.934
18	0.819	0.815	0.808	0.727	0.722	0.725	0.804	**0.842**	0.798	0.747	0.823
19	0.958	0.954	0.955	0.907	0.913	0.917	0.956	0.963	0.951	0.967	**0.980**
20	0.750	0.753	0.743	0.638	0.676	0.659	0.701	0.788	0.708	0.723	**0.799**
21	0.742	0.751	0.721	0.554	0.563	0.561	0.763	0.783	0.733	0.691	**0.796**

**Table 16:** Average selection size with various datasets for the compared algorithms with the three different initialization methods.

No.	WOA	bWOA-S	bWOA-v	BALO1	BALO2	BALO3	PSO	bGWO	bDA	bSSA	bSSARM-SCA
1	0.60886	0.63861	0.56741	0.47538	0.50271	0.50868	0.63800	0.63864	0.50675	0.58513	**0.39845**
2	0.77512	0.97084	0.75567	0.61843	0.63723	0.62091	0.52900	0.79141	0.80453	0.71853	**0.41776**
3	0.66136	0.76385	0.60682	0.62082	0.61776	0.62537	0.60000	0.59196	**0.47186**	0.59521	0.59631
4	0.62575	0.69964	0.58316	0.55874	0.56187	0.54282	0.64400	0.58202	**0.47052**	0.59542	0.58343
5	0.64672	0.73931	0.59172	0.54452	0.59815	0.56953	0.56500	0.62820	0.45986	0.58241	**0.43273**
6	0.64745	0.66347	0.55684	0.60581	0.60552	0.62386	0.52700	0.62140	0.43832	0.59984	**0.39566**
7	0.60278	0.66867	0.59251	0.54545	0.55643	0.54014	0.56100	0.61205	**0.40635**	0.59132	0.41883
8	0.55591	0.54555	0.54181	0.51756	0.45773	0.47854	0.61300	0.57510	0.41768	0.49586	**0.40025**
9	0.53255	0.58447	0.54674	0.50805	0.52583	0.50498	0.42600	0.62824	0.44291	0.52230	**0.41532**
10	0.70463	0.90372	0.67915	0.61943	0.62585	0.62323	0.57500	0.76343	**0.53323**	0.72852	0.67424
11	0.73333	0.90562	0.70748	0.62677	0.63145	0.63089	0.75300	0.79986	**0.58678**	0.73515	0.70038
12	0.64096	0.72682	0.69783	0.51643	0.54204	0.54291	0.47900	0.62248	0.61836	0.62253	**0.45673**
13	0.49951	0.46766	0.61592	0.39480	0.40371	0.44680	0.47400	0.42963	**0.17891**	0.47631	0.25214
14	0.72478	0.87848	0.69151	0.62203	0.60811	0.62151	0.69600	0.76442	0.63473	0.74125	**0.59421**
15	0.66752	0.74691	0.60294	0.59162	0.56681	0.61087	0.52100	0.61076	**0.37889**	0.59847	0.46316
16	0.57203	0.62386	0.60253	0.51881	0.49581	0.51047	0.55800	0.60791	0.48891	0.55274	**0.45746**
17	0.66791	0.79945	0.59723	0.62171	0.62593	0.62368	0.85900	0.64188	**0.50080**	0.65842	0.52873
18	0.69274	0.79430	0.58856	0.62131	0.61927	0.62394	0.65300	0.64942	0.48531	0.65752	**0.47841**
19	0.66856	0.77001	0.57541	0.62471	0.62492	0.62782	0.78200	0.68587	0.48756	0.64968	**0.47485**
20	0.66241	0.72775	0.60096	0.60554	0.58972	0.59054	0.49700	0.62543	0.50483	0.62430	**0.47124**
21	0.64848	0.71164	0.53685	0.62125	0.62182	0.62323	0.55300	0.49162	0.47485	0.51429	**0.46991**

**Table 17:** Classification accuracy of the proposed bSSARM-SCA method and three recent ISSA variants for the 21 utilized datasets.

	Small Initialization				Large Initialization				Mixed Initialization			
**No.**	**bSSARM-SCA**	**bISSA1**	**bISSA2**	**bISSA3**	**bSSARM-SCA**	**bISSA1**	**bISSA2**	**bISSA3**	**bSSARM-SCA**	**bISSA1**	**bISSA2**	**bISSA3**
1	**0.974**	0.956	0.962	0.935	**0.948**	0.949	0.951	0.926	**0.995**	0.959	0.974	0.942
2	**0.780**	0.769	0.771	0.762	**0.814**	0.796	0.801	0.785	**0.826**	0.802	0.806	0.793
3	**0.665**	0.652	0.649	0.638	**0.920**	0.897	0.886	0.869	**0.987**	0.962	0.944	0.926
4	**0.343**	0.310	0.324	0.298	**0.094**	0.085	0.081	0.079	**0.079**	0.074	0.072	0.068
5	**0.754**	0.728	0.739	0.720	**0.714**	0.696	0.698	0.653	**0.694**	0.680	0.682	0.647
6	**0.915**	0.893	0.910	0.885	**0.794**	0.772	0.785	0.751	**0.789**	0.769	0.774	0.748
7	0.874	0.859	**0.891**	0.851	0.856	0.843	**0.863**	0.836	0.894	0.887	**0.902**	0.879
8	**0.831**	0.817	0.820	0.803	**0.715**	0.702	0.705	0.698	**0.795**	0.781	0.786	0.769
9	**0.938**	0.914	0.912	0.906	**0.645**	0.628	0.623	0.615	**0.611**	0.602	0.601	0.594
10	0.916	0.908	**0.917**	0.902	**0.918**	0.911	0.917	0.909	0.971	0.956	**0.973**	0.949
11	**0.830**	0.814	0.811	0.809	**0.830**	0.813	0.809	0.806	**0.851**	0.837	0.835	0.825
12	**0.712**	0.709	0.709	0.705	**0.707**	0.703	0.704	0.699	**0.696**	0.690	0.691	0.682
13	0.764	0.756	**0.771**	0.753	0.786	0.779	**0.792**	0.772	0.776	0.768	**0.789**	0.761
14	**0.953**	0.942	0.937	0.933	**0.886**	0.863	0.866	0.857	**0.910**	0.902	0.898	0.883
15	**0.935**	0.916	0.919	0.904	**0.931**	0.909	0.914	0.896	**0.893**	0.868	0.872	0.859
16	**0.977**	0.967	0.963	0.958	**0.898**	0.884	0.879	0.871	**0.956**	0.941	0.936	0.928
17	0.914	**0.918**	0.915	0.904	0.933	**0.936**	0.926	0.917	0.934	**0.938**	0.928	0.925
18	**0.891**	0.863	0.869	0.858	**0.861**	0.837	0.842	0.831	**0.823**	0.806	0.809	0.793
19	**0.904**	0.897	0.901	0.884	**0.978**	0.956	9.971	0.948	**0.980**	0.968	0.973	0.962
20	0.878	**0.882**	0.866	0.859	0.752	**0.761**	0.735	0.728	0.799	**0.806**	0.783	0.775
21	0.894	0.873	**0.899**	0.865	0.793	0.784	**0.804**	0.779	0.796	0.789	**0.814**	0.787
